# Cooperative phototherapy based on bimodal imaging guidance for the treatment of uveal melanoma

**DOI:** 10.1186/s12951-023-01891-6

**Published:** 2023-05-04

**Authors:** Tong Huang, Xinzhi Xu, Chen Cheng, Jianxin Wang, Liping Yang

**Affiliations:** 1 Chongqing Key Laboratory of Ultrasound Molecular Imaging, The Second Affiliated Hospital of Chongqing Medical University, Chongqing, 400010 P. R. China; 2 Department of Ultrasound, Chongqing University Cancer Hospital, Chongqing, 400030 P. R. China; 3 Department of Ultrasound, The First Affiliated Hospital of Harbin Medical University, Harbin, 150001 P. R. China; 4 Department of Laboratory Medicine, The Second Affiliated Hospital of Chongqing Medical University, Chongqing, 400010 P. R. China

**Keywords:** Uveal melanoma, Photothermal therapy, Photodynamic therapy, Dual-modal imaging, Nanomedicine

## Abstract

**Background:**

Uveal melanoma (UM) is adults’ most common primary intraocular malignant tumor, prone to metastasis and high mortality. Eyeball enucleation commonly used in the clinic will lead to permanent blindness and mental disorders. Thus, new methods are urgently needed to diagnose and treat UM early to preserve patients’ vision.

**Methods and results:**

Herein, multifunctional nanoparticles (NPs) were synthesized by loading chlorin e6 (Ce6) in poly-lactic-co-glycolic acid (PLGA) NPs and wrapping Fe^III^-tannic acid (Fe^III^-TA) on the outside (Fe^III^-TA/PLGA/Ce6, designated as FTCPNPs). Then, the synergistic photothermal therapy (PTT) and photodynamic therapy (PDT) antitumor effects of FTCPNPs excited by near-infrared (NIR) laser were evaluated. Moreover, we verified the mechanism of synergistic PTT/PDT leading to mitochondrial dysfunction and inducing tumor cell apoptosis. Additionally, FTCPNPs can be used as excellent magnetic resonance (MR)/photoacoustic (PA) imaging contrast agents, enabling imaging-guided cancer treatment. Finally, The NPs have good biological safety.

**Conclusion:**

This noninvasive NIR light-triggered cooperative phototherapy can easily penetrate eye tissue and overcome the disadvantage of limited penetration of phototherapy. Therefore, cooperative phototherapy is expected to be used in fundus tumors. This treatment model is applied to UM for the first time, providing a promising strategy and new idea for integrating the diagnosis and treatment of UM.

**Supplementary Information:**

The online version contains supplementary material available at 10.1186/s12951-023-01891-6.

## Background

The most common intraocular malignancy in adults is uveal melanoma (UM)[1]. Asia has a much lower incidence of UM than Europe, North America and Australia [2]. The prevalence in males is higher than that in females [3]. UM patients often have exophthalmos, soft periocular tissue edema, intraocular hemorrhage, and eye movement disorders [4]. The size of the melanoma is also correlated with the outcome: each millimeter of increased thickness added a 5% risk of metastasis at ten years [5], leading to an abysmal prognosis [6]. The metastatic disease affects nearly half of the patients, usually involving the liver and is typically fatal within one year [7]. Currently, no standard therapeutic strategy for preventing or treating metastases [8]. The purpose of treating intraocular tumors is to protect the eyeball, preserve vision and prolong the life of patients. Surgical resection is performed as the primary treatment for UM [9]. However, permanent blindness and severe facial disfigurement caused by this measure can easily lead to severe mental disorders [10]. Therefore, providing accurate imaging evidence for diagnosis and prompt medical intervention in the early stage is the primary task of UM treatment.

Currently, the primary imaging examinations of UM are diagnostic methods that include clinical study (ophthalmoscopy, biomicroscopy and ultrasound)[7]. Although there are a variety of ophthalmic imaging techniques, traditional optical imaging and ultrasound imaging cannot effectively detect early lesions of UM [11]. Moreover, magnetic resonance imaging (MRI) has a significant role in confirming the diagnosis and assessing the local extent of UM, which affects treatment planning and follow-up after radiotherapy [12]. However, a single imaging method has some shortcomings in diagnosing UM early. As a new imaging mode, photoacoustic imaging (PAI) can noninvasively evaluate endogenous tissue with optical contrast and unique spatiotemporal resolution. It has high resolution and image contrast advantages and far-reaching clinical significance for cancer diagnosis [13]. Therefore, finding a sensitive and accurate multimodal imaging method to diagnose UM is urgent.

In recent years, nanomedicine has gradually become popular in anticancer research with new concepts such as integrating diagnosis and treatment and precision treatment [14]. With the advantages of a high drug loading rate and therapeutic specificity, nanomaterials have been widely used to effectively deliver drugs [15], small molecules [16], peptides, and nucleic acids in treating all kinds of tumors [17]. Compared with traditional radiotherapy and chemotherapy, phototherapy mediated by nanoparticles (NPs), such as photothermal therapy (PTT) and photodynamic therapy (PDT), has the advantages of being noninvasive, having high spatiotemporal selectivity and low systemic toxicity [18]. It has gradually become a promising cancer treatment strategy, and the continuous update of nanotechnology has given great potential for the clinical transformation of phototherapy. PTT uses photothermal agents (PTAs) [19] to convert light energy into heat energy [19]. It uses the rise of temperature to induce cancer cell death [20]. However, most existing phototherapy limits its clinical application due to the low penetration depth of infrared light to the tissue [21]. Because of the unique transparency of eye tissue, laser therapy is a relatively mature method in fundus disease treatment, and light can easily penetrate the tissue to reach deeper areas. Therefore, phototherapy has a broad prospect in the field of fundus diseases. In recent years, gold NPs have shown great potential in ophthalmic diagnosis and treatment [22], it offers considerable photothermal therapeutic effect under the excitation of near-infrared (NIR). However, its application prospect in ophthalmology is limited by many factors, such as the high price of gold NPs, complex synthesis steps, limited photothermal conversion efficiency, poor biocompatibility, etc. On the other hand, this therapy’s therapeutic effect is remarkable. A deficiency of hyperthermia or uneven heat distribution within the tumor region may lead to tumor regrowth [23]. Alternatively, PDT can produce toxic reactive oxygen species (ROS) under the excitation of specific wavelengths, destroy the function of mitochondria and induce apoptosis of tumor cells [24]. However, PDT is severely limited in effectiveness due to its high dependence on oxygen. When applied separately, both phototherapy techniques present inherent limitations [25]. Many studies have shown that the fusion of PTT and PDT into a single nanoplatform can complement each other and show better synergistic antitumor effects [26]. In addition, a double laser combined with PTT and PDT can improve the therapeutic efficiency of the tumor area and increase the depth of penetration [27], which is much stronger than monotherapy [28].

Various PTAs have been widely used to evaluate eye diseases [22]. The photosensitizer chlorin e6 (Ce6) has good biosafety and photodynamic effect, but like most photosensitizers, hydrophobic Ce6 is easy to aggregate in an aqueous solution. As a drug carrier, poly-lactic-co-glycolic acid (PLGA) is characterized by hydrophobicity and high drug loading. It has been approved by the Food and Drug Administration (FDA) [29]. The Ce6 was encapsulated in PLGA and used in PDT under 660 nm NIR light excitation [30]. Fe^III^-tannic acid (Fe^III^-TA), a new class of hybrid materials combining organic and inorganic components, can diagnose and treat tumors using iron ions and multiple functions [31]. Compared with the previously reported study of gold NPs, Fe^III^-TA has the advantage of a novel structure, simple synthesis process, low cost and better safety. Fe^III^-TA also showed excellent photothermal performance under excitation with 808 nm laser [32]. In addition, its excellent photothermal conversion efficiency determines the signal intensity of PAI, and paramagnetism can be used as MRI contrast agents to provide precise and personalized interventions for cancer therapy.

In our work, we used PLGA to carry Ce6 and coated Fe^III^-TA on the surface of PLGA. The FTCPNPs can enter tumor through trans-endothelial pathways [33]. Under 808 nm and 660 nm laser excitation, the local irradiated site warmed up and produced toxic singlet oxygen (^1^O_2_). Finally, the UM was ablated by PTT and PDT. Next, we discussed the therapeutic mechanism of collaborative phototherapy. Apoptosis of C918 cells induced by mitochondrial-related dysfunction before and after treatment was detected by RNA-sequencing. In addition, the best time window for NPs to enter the tumor was determined by PAI/MRI modes. In vivo experiments showed that synergistic phototherapy can inhibit tumor growth and recurrence. It is worth pointing out that the eyeball is the only transparent tissue in the human body and the NIR laser can easily penetrate the eye tissue; the disadvantage of limited penetration of phototherapy can be avoided. Therefore, inspired by nanomedicine, the overall design of the Fe^III^-TA/PLGA/Ce6 nanoparticles (FTCPNPs) is highly coordinated and has a simple synthesis process. It is possible to detect tumors early with these nanoplatforms, adjust therapeutic time windows, monitor treatment processes, and optimize treatment outcomes, providing a novel approach for integrating diagnosis and treatment of UM.

**Scheme 1:**
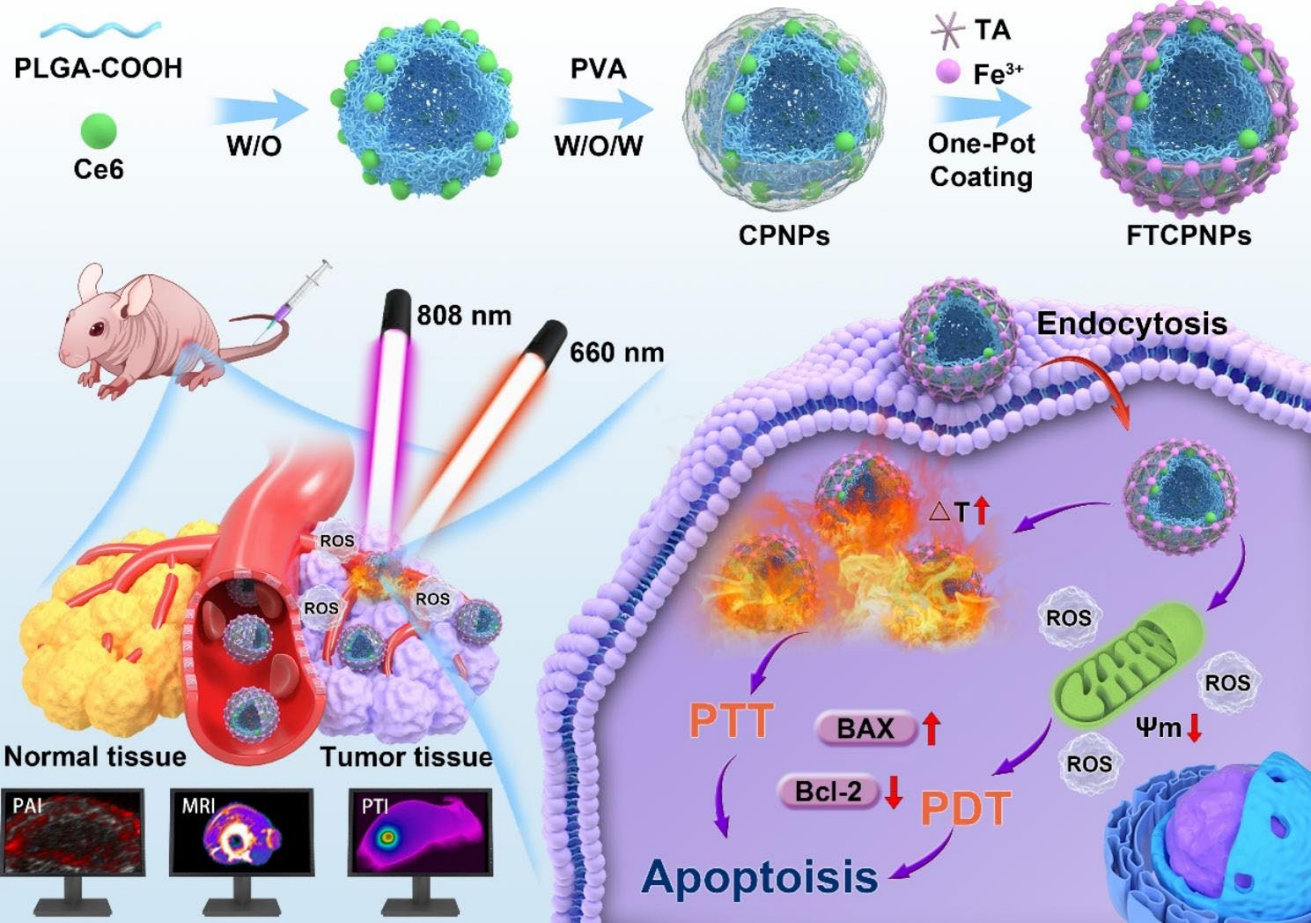
FTCPNPs synthesis and combination effects of PTT/PDT against tumors

## Materials and methods

### Reagents

The poly lactic-co-glycolic acid (PLGA) (50:50, MW: 12,000 Da) with a carboxylic acid (-COOH) end group which was purchased from Daigang BIO Engineer Co., Ltd. (Shan Dong, China). Ferric chloride hexahydrate (FeCl_3_·6H_2_O), tannic acid (TA), and polyvinyl alcohol (PVA, MW: 25,000 Da) were purchased from Sigma-Aldrich Chemical Co. (St. Louis, MO, USA). Chlorin e6 (Ce6, purity by HPLC ≥ 90%) was purchased from Meilunbio (Dalian, China). Other reagent kits: singlet oxygen sensor green (SOSG) (Invitrogen, Massachusetts, USA). Calcein-AM (CAM), propidium iodide (PI), and CCK-8 assays were purchased from Dojindo Laboratories (Kumamoto, Japan). JC-1 assay kit, 2′,-7′-dichlorodi-hydrofluorescein diacetate (DCFH-DA), 4,6-diamidino-2-phenylindole (DAPI) were all purchased from Beyotime Biotechnology Ltd., Co. (Shanghai, China).

### Synthesis of FTCPNPs

First, PLGA (50 mg) was dissolved in 2 mL dichloromethane (CH_2_Cl_2_), Ce6 solution (0.5 mL, 5 mg/mL dissolved in methanol) was added, and the mixture was placed in an ultrasonic cleaning instrument for 5 min and completely dissolved. After that, ultra-pure water (200 µL) was added, and the probe sonicator (Sonics & Materials, Inc., USA) was used to emulsify the mixture for 3 min at an intensity of 45 W. For the second emulsion, 4% PVA solution (8 mL) was added to the above-emulsified solution and homogenized using the same sonicator at an intensity of 35 W for 3 min. After that, 2% isopropanol solution (10 mL) was added and stirred with a magnetic agitator for 3 h to remove CH_2_Cl_2_ in a well-ventilated fume hood. Then, the samples were centrifuged for 5 min (7,058 × g) and washed with ultra-pure water 3 times to obtain PLGA/Ce6 nanoparticles (CPNPs).

Finally, CPNPs (1 mL, 0.5 mg/mL) were added to TA solution (5 µL, 40 mg/mL) and FeCl_3_ solution (5 µL, 10 mg/mL) in turn, placed in a vortex apparatus for 15 s to mix evenly and added NaOH aqueous solution (7.5 µL, 0.1 M) to adjust pH to 7.0. Finally, centrifuge and wash 3 times with ultrapure water. The FTCPNPs were complete.

### Characterization of FTCPNPs

Transmission electron microscope (TEM, Hitachi-7500, Japan) and scanning electron microscopy (SEM, Thermo Apreo S HiVac FEI, USA) were utilized to analyze the morphology and structure of the FTCPNPs. An FEI-Talos F200S electron microscope was used to confirm the presence of Fe in FTCPNPs using area-elemental mapping. Dynamic light scattering (DLS, Brookhaven, USA) was used to determine the size distributions and zeta potentials. The colloidal stability of FTCPNPs dissolved in 5% glucose solution was monitored in 7 d, respectively. A confocal laser scanning microscope (CLSM, A1, Nikon, Tokyo, Japan) was applied to observe the fluorescence of FTCPNPs. The absorption spectrum of FTCPNPs, CPNPs, Fe^III^-TA, free Ce6, and PLGANPs aqueous solutions was measured by UV-Vis spectrometer (Shimadzu, UV-3600, Japan) to observe the presence of Ce6 and Fe^III^-TA in the FTCPNPs. To detect the UV-Vis absorption spectrum of Ce6 and TA. Then the inductively coupled plasma optical emission spectrometer (ICP-OES, Agilent 5110, USA) was used to detect the amount of Fe in FTCPNPs. After that, the encapsulation efficiency and loading capacity of Ce6, TA and Fe were calculated (Supporting Information for details). Magnetization hysteresis loops of FTCPNPs were detected by vibrating sample magnetometer (VSM, LakeShore7404, USA).

### In vitro photothermal effects

Different concentrations (0.25, 0.5, 0.75, 1.0, and 1.25 mg/mL) of 200 µL FTCPNPs aqueous solution were added to 96-well plates to measure their thermal profiles under 808 nm laser (2.0 W/cm^2^, 10 min, the laser spot size: 1.0 cm^2^) illumination. The concentration of 1.25 mg/mL FTCPNPs was irradiated for 10 min at various intensities (2.0, 1.5, 1.0, and 0.5 W/cm^2^). To evaluate the practical components of photothermal properties, FTCPNPs aqueous solution (200 µL, 1.0 mg/mL), Fe^III^-TA, FeCl_3_, TA aqueous solution and free Ce6 with identical concentration were added to 96-well plates, and the thermal profiles of these samples were measured under irradiation with 808 nm laser at 2.0 W/cm^2^ for 10 min. An infrared thermal imaging camera (Fotric 226, Shanghai, China) was utilized to record the temperature change over time. To detect the photothermal stability of FTCPNPs, the FTCPNPs aqueous solution was irradiated by 808 nm laser for repeated heating and cooling cycles. The photothermal conversion efficiency was calculated for 10 min continuous irradiation with FTCPNPs (200 µL, 1 mg/mL).

### Cell culture and establishment of tumor-bearing animal model

The human choroid melanoma cells (C918) and adult retinal pigment epithelial cell line-19 (ARPE-19) were purchased from Procell Life Science&Technology Co., Ltd (Wuhan, China). C918 and ARPE-19 cells were cultured in RMPI-1640 medium containing 10% FBS and 1% penicillin/streptomycin in an incubator at 37 °C and 5% CO_2_. Healthy male nude mice (6–8 weeks old) were obtained from the Beijing HFK Bioscience Co., Ltd. The Animal Ethics Committee approved all the animal experiments at Chongqing Medical University.

To establish C918 tumor xenograft, 100 µL (3 × 10^6^ C918 cells suspended in PBS solution) suspension was injected into each mouse’s subcutaneous tissue of the root of the right thigh.

### Cellular uptake behaviors of FTPCPNPs

C918 cells (5 × 10^4^ per dish) were seeded in confocal laser scanning microscopy (CLSM) dishes for 24 h and co-incubated with FTCPNPs (1 mL, 10 µg/mL, the fluorescence signal comes from Ce6) for different time points (0.5, 1, 3 and 6 h) to observe intracellular uptake. Then, fresh RMPI-1640 was used to wash the confocal dishes 3 times, and 4% paraformaldehyde (1 mL) was added to fix the C918 cells for 15 min and dyed with DAPI (200 µL) for 8 min. Finally, cellular uptake was observed by CLSM. Flow cytometry (BD FACSVantage SE, USA) was used to determine the intracellular uptake of FTCPNPs at various time points.

### Determination of ROS levels

In vitro ROS levels were measured using SOSG. As a brief overview, different concentrations of FTCPNPs and SOSG (5 µM) were added to cuvettes and irradiated with 660 nm laser (Stone Laser, China) at a power density of 5 mW/cm^2^ (the laser spot size: 2.0 cm^2^) for different time intervals. A multimode reader (Shimadzu RF-5310PC, Japan) was used to observe changes in the intensity of SOSG fluorescence. Intracellular ROS levels were determined using the fluorescent probe 2′,-7′-dichlorodi-hydrofluorescein diacetate (DCFH-DA, λex/λem = 488 nm/525 nm). C918 cells were seeded in confocal dishes at a density of 5 × 10^4^ cells per dish. They were divided into 4 groups: control group (Control), laser only group (660 nm laser, Laser), FTCPNPs group (FTCPNPs), FTCPNPs + 660 nm laser group (FTCPNPs + Laser). After 24 h of incubation, the cells of FTCPNPs and FTCPNPs + laser groups were co-incubated with FTCPNPs at the same Ce6 concentration of 8 µg/mL. After 8 h of co-incubation with the corresponding FTCPNPs, the dishes were rinsed with fresh RMPI-1640, and DCFH-DA was added staining for 20 min. Cells in the laser and FTCPNPs + laser group were irradiated with 660 nm laser (5 mW/cm^2^, 3 min). Excess dye was washed away with PBS. Intracellular ROS levels were observed by CLSM. Flow cytometry was used to analyze the collected cells.

### Synergistic therapeutic effects in vitro

First, the safety performance of FTCPNPs was estimated by CCK-8 assay. Typically, C918 and ARPE-19 cells were seeded in 96-well plates at a concentration of 1 × 10^4^ cells per well for 24 h. Then, different concentrations of FTCPNPs (0, 0.0625, 0.125, 0.25, 0.5, 1, 2 mg/mL) were added followed by 48 h of incubation. After co-incubation with 10 µL of CCK-8 solution for 45 min, cytotoxicity was detected. Finally, at an absorbance of 450 nm, the viability of the cells was determined using a microplate reader.

C918 cells were seeded in 96-well plates at a density of 1 × 10^4^ cells per well for 24 h. These cells were randomly divided into the following groups: control group (Control), laser only group (660 nm + 808 nm laser, Laser), FTCPNPs group (FTCPNPs), FTCPNPs + 808 nm laser (2.0 W/cm^2^, 5 min) group (PTT), FTCPNPs + 660 nm laser (5 mW/cm^2^, 3 min) group (PDT), FTCPNPs + 808 nm and 660 nm laser group (PTT/PDT). The cells of FTCPNPs, PTT, PDT, and PTT/PDT groups were co-incubated with FTCPNPs at the concentration of 0.5 and 1.0 mg/mL. As described above, different treatments were applied after 8 h of co-incubation. Finally, the C918 cell viability was assessed using a microplate reader at an absorbance of 450 nm after 45 min of co-incubation with 10 µL of CCK-8 solution.

Similarly, live/dead cell staining was used to assess the antitumor efficacy in vitro. C918 cells were seeded into 6-well plates (2 × 10^5^ cells per well) for 24 h, then co-incubated with FTCPNPs (0.5 mg/mL) for 8 h. After that, C918 cells were treated according to different groups: they were further irradiated sequentially with 808 nm laser (2.0 W/cm^2^, 5 min) at room temperature and/or a 660 nm laser (5 mW/cm^2^, 3 min) in an ice bath (to avoid PTT effects). Finally, to quantify live/dead cells by CLSM, Calcein-AM (CAM, 2 µM)/PI (propidium iodide, 4 µΜ) dye solution was used. Different groups of cells were treated in the same way and incubated with annexin V-FITC/PI for 20 min before conducting flow cytometry analysis.

### Detection of mitochondrial depolarization

Mitochondrial membrane potential (MMP, Ψm) changes were monitored using the mitochondrial dye JC-1. First, C918 cells were seeded into confocal dishes at a density of 5 × 10^4^ cells per dish, incubated for 24 h, and then co-incubated with FTCPNPs for 8 h. The C918 cells were divided into the following groups: control group (Control), laser only group (Laser), FTCPNPs group (FTCPNPs), FTCPNPs + 808 nm laser group (PTT), FTCPNPs + 660 nm laser group (PDT), FTCPNPs + 808 nm and 660 nm laser group (PTT/PDT), and each group was treated separately. In the positive control group, cells were treated for 15 min with MMP inhibitor carbonyl cyanide-m-chlorophenylhydrazone (CCCP). As a next step, JC-1 probe staining solution was added to fresh medium containing cells of each group for 20 min in the dark and observed by CLSM. Flow cytometry was used to quantify the levels of MMP in cells treated as described above.

### RNA-sequencing and bioinformatics analysis

C918 cells were seeded into T25 cell culture bottles (1 × 10^6^ cells per bottle) for 24 h. The PTT/PDT group was co-incubated with FTCPNPs for 8 h. Then, the C918 cells were treated with 808 nm and 660 nm laser. Wash off the culture medium with PBS. C918 cells collected by centrifugation dissolved rapidly in TRIzol cleavage. We used DNBSEQ-T7 (MGI Tech Co., Ltd, China) to perform RNA-sequencing.

### Western-blot

The C918 cells were co-incubated with FTCPNPs for 8 h, and the cell culture conditions were similar to those of the cytotoxicity test. For the extraction of total protein, the C918 cells were collected and then lysed on ice in a RIPA buffer containing 1% phenyl methane sulfonyl fluoride (PMSF) for 30 min. A BCA protein assay kit was used to determine the protein concentration. 10% SDS-PAGE gels were used to separate the proteins, which were then transferred onto a polyvinylidene fluoride (PVDF) membrane. The membranes were incubated with specific antibodies (Bax antibody 1: 10,000, Bcl-2 antibody 1: 1000) overnight at 4 ℃. Following blocking with 5% skim milk for 1 h, the membrane was incubated with a secondary antibody (HRP conjugated goat anti-rabbit IgG) for 1 h at room temperature. An enhanced chemiluminescence system (Pierce, USA) was used to visualize the membranes.

### MR/PA imaging and bio-distribution of FTCPNPs

MRI in vitro, the prepared FTCPNPs aqueous solution at concentrations of 0.0375, 0.075, 0.15, 0.3, 0.6, and 1.2 mg/mL were placed in plastic tubes. An MRI system (Siemens Medical System, Chongqing People’s Hospital) provided the corresponding images at 3.0 T with a gradient echo sequence, and the corresponding T1 relaxation time was obtained. The MRI parameters were as follows: a gradient echo sequence (repetition time (TR)/echo time (TE)) of 790/11 ms, and a slice thickness of 2.00 mm. PAI in vitro, serial concentrations of 0.25, 0.5, 0.75, 1.0, and 1.25 mg/mL were used to detect PA signals and evaluate linearity as a function of FTCPNP concentrations. VEVO LAZR PA imaging system (FUJIFILM Visual Sonics, Inc, Canada) was used to obtain the PA images. To evaluate the accumulation of FTCPNPs in tumors, T1-weighted MRI and PAI (λex = 690 nm) of C918-tumor-bearing mice were performed. FTCPNPs solution (200 µL, 5 mg/mL) was injected into the tail vein, and the corresponding MR and PA images were collected at different time points (4, 8, 24, 48 h).

FTCPNPs were labeled with 1,1′-dioctadecyl-3,3,3′,3′-tetramethylindotricarbocyanine iodide (DiR) and injected into C918-tumor-bearing nude mice through the tail vein. Fluorescence images were obtained using a fluorescence system (λex/λem = 740 nm/790 nm) at different time points (2, 12, 24, and 48 h). Finally, tumors and major organs of mice were imaged with fluorescence, and the related fluorescence signals were analyzed.

### In vivo anticancer efficacy under PTI guidance and biosafety of FTCPNPs

C918-tumor-bearing nude mice were randomly divided into 6 groups (n = 6) after the tumor volume reached 60 mm^3^ after intravenous injection of FTCPNPs: (a) control group (Control), (b) laser only group (Laser), (c) FTCPNPs group (FTCPNPs), (d) FTCPNPs + 808 nm laser group (PTT), (e) FTCPNPs + 660 nm laser group (PDT), (f) FTCPNPs + 808 nm and 660 nm laser group (PTT/PDT). Mice in (a) injected with 200 µL 5% glucose solution were set as the control group. Mice in (c) (d) (e) (f) were injected with FTCPNPs (200 µL, 5 mg/mL). For group (d), the tumors were irradiated with an 808 nm laser (2.0 W/cm^2^, 10 min) at 24 h post-injection. For group (e), the tumors were irradiated with a 660 nm laser (95 mW/cm^2^, 10 min), and for the group (b) and (f) the tumors were irradiated with 808 nm and 660 nm laser for 10 min. To avoid PTT effects during treatment, an infrared thermal camera was used to monitor temperature variations in the group (e). Each mouse’s tumor volume and body weight were recorded every 2 d during a 14 d observation period. The tumor volume was calculated as follows: V = length × width^2^/2. Tumor volume change was represented by V/V_0_ (V_0_ was set as the initial tumor volume). All PDT processes were performed immediately after PTT. In each group, one mouse was sacrificed 24 h after the treatment. For hematoxylin and eosin (H&E) staining, tumors, liver, spleen, lung, and kidney were harvested and preserved in paraformaldehyde at 4%. Transferase-mediated dUTP nick-end labeling (TUNEL) and proliferating cell nuclear antigen (PCNA) staining were performed on tumor tissues to determine tumor proliferation. In addition, the tumor tissues were stained with Bax and Bcl-2 immunofluorescence.

### In vivo evaluation of the toxicity of FTCPNPs

FTCPNPs (200 µL, 5 mg/mL) were injected into healthy Kunming mice (n = 25, 6 weeks). In the control group (n = 5, 6 weeks), mice were injected with 5% glucose solution. After a specified time (1, 3, 7, 14, and 28 d post-injection), the mice were euthanized and their blood was collected for blood biochemistry and routine blood examinations. Major organs of mice were removed and fixed with 4% polyoxymethylene before H&E staining.

### Statistical analysis

All data are expressed as the mean ± standard deviation (SD), and the significance of differences among groups was evaluated with one-way ANOVA and Student’s t-test (*p < 0.05, **p < 0.01, ***p < 0.001).

## Results and discussion

### Synthesis and characterization of FTCPNPs

**Fig. 1:**
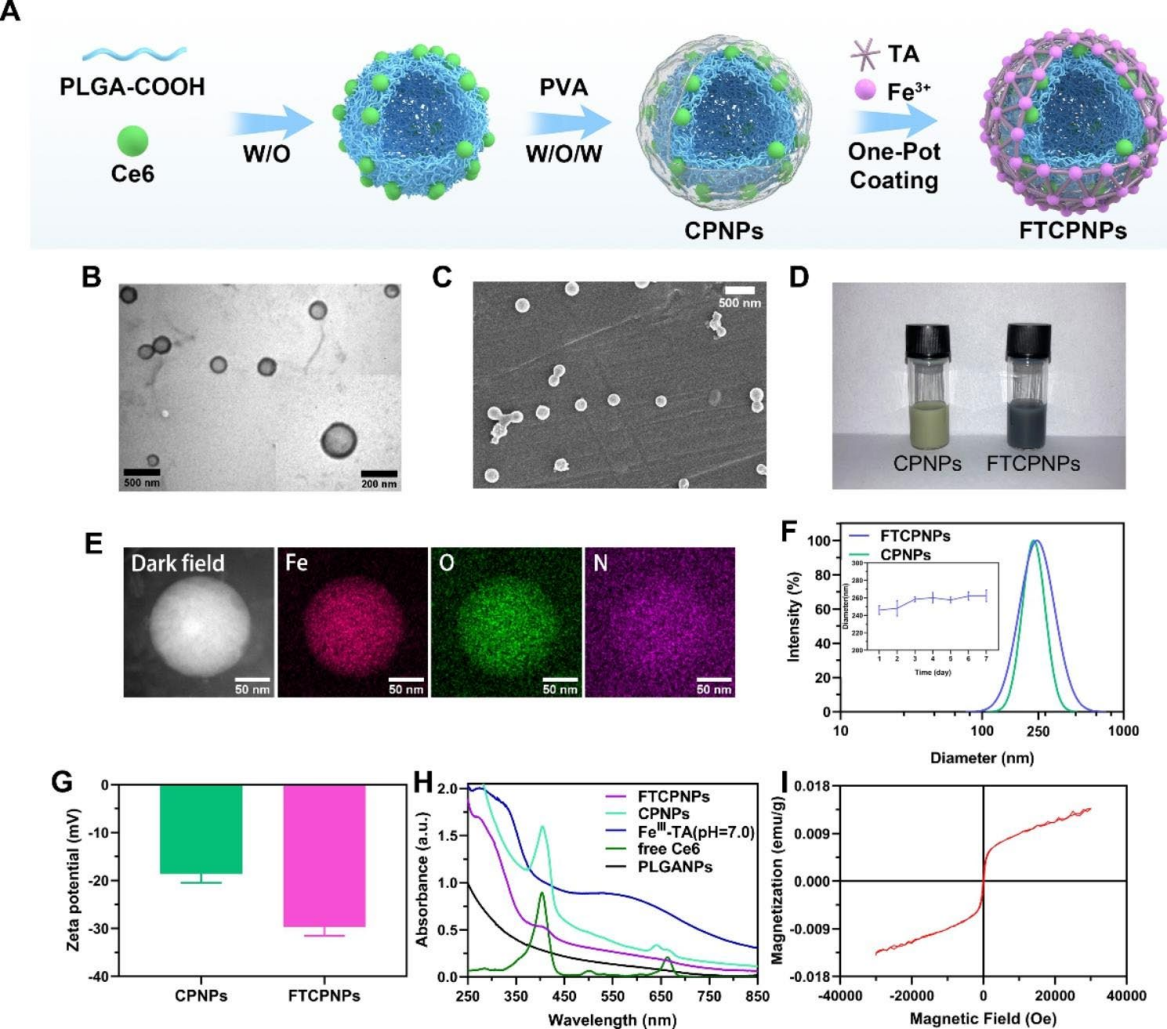
**A** Schematic diagram for the fabrication of FTCPNPs. **B** Low and high magnification TEM images of FTCPNPs. Scale bar: 500 nm and 200 nm. **C** SEM image of FTCPNPs. Scale bar: 500 nm **D** The appearance of CPNPs and FTCPNPs aqueous solution. **E** Elemental distribution mappings of FTCPNPs. Scale bar: 50 nm. **F** Size distribution of FTCPNPs and the change of average size with prolonged time duration (n = 3). **G** Zeta potential of CPNPs and FTCPNPs (n = 3). **H** UV-Vis spectrum of FTCPNPs, CPNPs, Fe^III^-TA, free Ce6, and PLGANPs. **I** Magnetization hysteresis loop of FTCPNPs ranging from − 30 kOe to + 30 kOe at 300 K

PLGA was used to carry the hydrophobic material Ce6 in the shell using a double-emulsion approach to obtain CPNPs. Fe^III^-TA was formed through the coordination reaction between the natural polyphenol TA as an organic ligand and Fe^III^, and then wrapped around CPNPs in a one-step coating process (Fig. 1A). FTCPNPs were obtained. The appearance of the CPNPs aqueous solution changed from green to dark purple when Fe^III^-TA was loaded (Fig. 1D), and the FTCPNPs had a clear core-shell structure as shown by TEM (Fig. 1B). These results demonstrated that Fe^III^-TA was loaded successfully. FTCPNPs showed spherical morphology by SEM (Fig. 1C). The high-angle annular dark-field (HAADF) STEM image of FTCPNPs also showed a spherical shape. The area elemental mapping analysis revealed the presence of the Fe element, which also illustrated the successful loading of Fe in FTCPNPs (Fig. 1E). Furthermore, the FTCPNPs’ red fluorescence (from Ce6) was detected by CLSM (Fig. S1A).

The average size of CPNPs was approximately 232.58 ± 5.73 nm. After being coated with Fe^III^-TA, the particle size increased to 246.32 ± 5.34 nm (Fig. 1F), in agreement with the size obtained by TEM. The low polydispersity (PDI) indices of FTCPNPs (0.086) and CPNPs (0.036) ensured the uniform dispersion of NPs. In addition, Fig. 1F showed nonsignificant diameter changes of FTCPNPs in 5% glucose solution during a 7 d storage period, and good stability of FTCPNPs was evident. The zeta potential of CPNPs was − 18.57 ± 1.875 mV. The Fe^III^-TA coated outside of CPNPs reduced the potential to − 29.70 ± 1.819 mV (Fig. 1G). In vivo, the negative zeta potential may facilitate nanodroplet repellence and prevent aggregation. Furthermore, the negative zeta potential NPs can prolong the circulation time of FTCPNPs in the blood by decreasing the clearance of the reticuloendothelial system (RES), which contributes to the delivery of these NPs.

UV spectroscopy revealed that both free Ce6 and CPNPs had an absorption peak at 404 nm. After coating with Fe^III^-TA, the UV absorption peak slightly redshifted to 402 nm and formed a slight and minor absorption peak. According to Fig. 1H, it was confirmed that the Ce6 photosensitizer was successfully loaded in FTCPNPs. In addition, the Ce6 encapsulation efficiency and loading capacity were 82.09 ± 2.332% and 4.561 ± 0.1296% according to the standard curve of Ce6 obtained by UV spectroscopy (Fig. S1B, C). Moreover, the encapsulation efficiency and loading capacity of TA were 26.35 ± 3.848% and 12.29 ± 1.796% according to the standard curve of TA obtained by UV spectroscopy (Fig. S1D). The encapsulation efficiency and loading capacity of Fe were determined to be 6.628 ± 0.3005% and 0.7732 ± 0.03505% according to the standard curve of Fe (Fig. S1E) by ICP-OES. The concentration of FTCPNPs corresponding to the concentration of Fe and Ce6 was calculated (Table S1). Finally, the magnetic properties of the FTCPNPs were determined by the magnetic hysteresis loop (Fig. 1I). Based on this study, it was confirmed that FTCPNPs display paramagnetic behavior, possibly arising from Fe^III^.

### In vitro photothermal effect

**Fig. 2:**
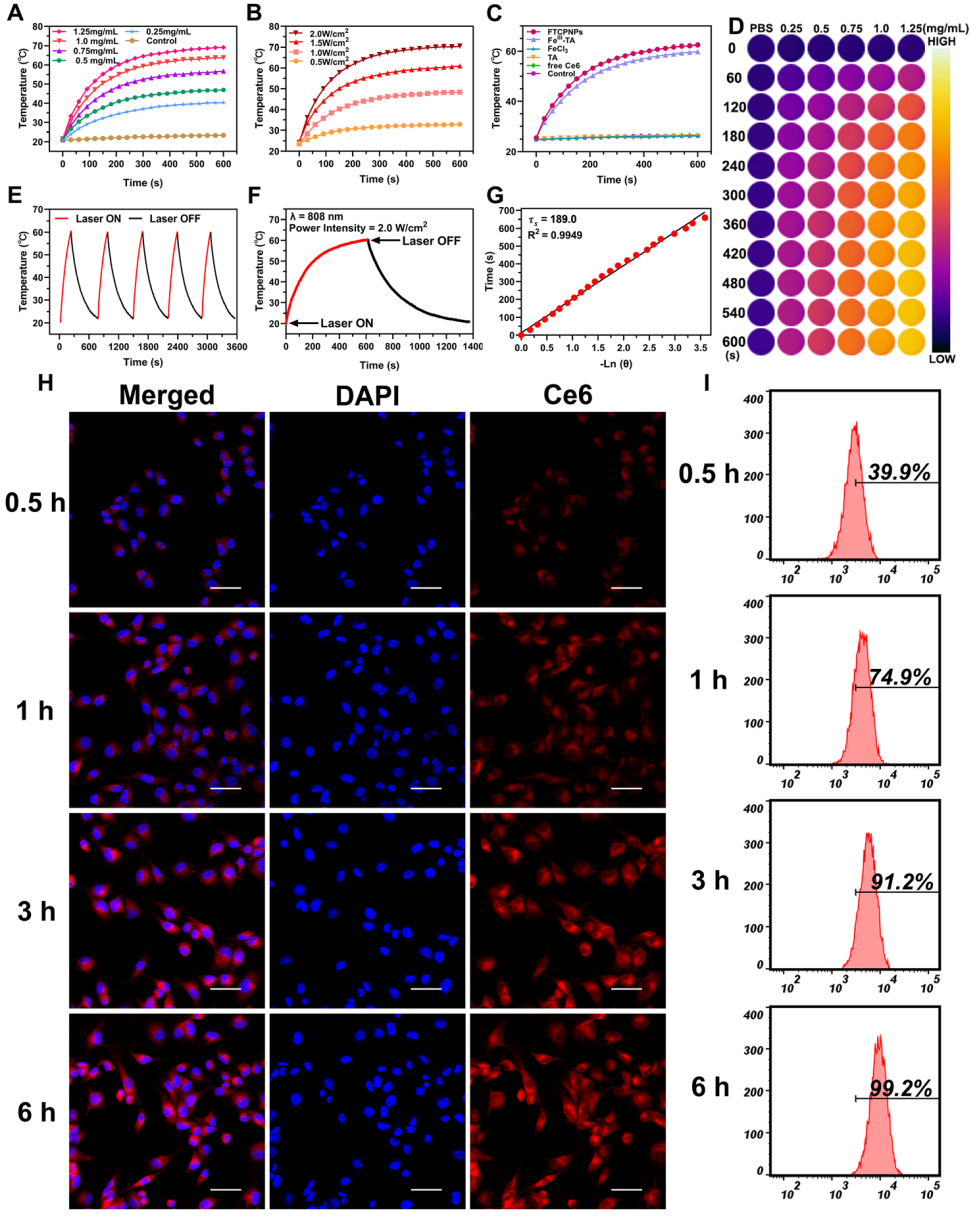
**A** At different concentrations (0.25, 0.5, 0.75, 1.0 and 1.25 mg/mL), photothermal curves of FTCPNPs aqueous solution with 808 nm laser irradiation (2.0 W/cm^2^). **B** FTCPNPs aqueous solution photothermal curves at increased power densities (0.5, 1.0, 1.5, and 2.0 W/cm^2^). **C** Different components of FTCPNPs and their photothermal performance. **D** Infrared thermal images of FTCPNPs irradiated with an 808 nm laser at different concentrations (0.25, 0.5, 0.75, 1.0, and 1.25 mg/mL). **E** FTCPNPs aqueous solution photothermal curves for 5 heating/cooling cycles. **F** FTCPNPs aqueous solution (1.0 mg/mL) was exposed to 808 nm laser irradiation (2.0 W/cm^2^) for 600 s followed by cooling. **G** Based on the cooling period, the time constant for heat transfer was calculated. **H, I** Intracellular uptake of FTCPNPs as observed by CLSM and quantified by flow cytometry analysis. Scale bar: 50 μm

To evaluate the photothermal performance of FTCPNPs, infrared thermal imaging cameras were used to measure temperature changes after 808 nm irradiation. After irradiation with 808 nm laser (2.0 W/cm^2^,10 min), the FTCPNPs aqueous solution showed a sharp temperature increase from 21.5 to 69.2 °C at the concentration of 1.25 mg/mL. The concentration-dependent and irradiation time-dependent relationship was observed for the various concentrations of FTCPNPs photothermal curves (Fig. 2A). Moreover, at the FTCPNPs concentration of 1.25 mg/mL, FTCPNPs aqueous solution also followed laser power-dependent photothermal performance, with an increase in laser power from 0.5 to 2.0 W/cm^2^, the maximum temperature rose from 32.9 to 70.5 °C after 10 min of exposure, respectively (Fig. 2B). Meanwhile, the photothermal imaging (PTI) performance of various FTCPNPs concentrations (Fig. 2D) and laser intensity (Fig. S2) were also presented. The results provided strong evidence of the efficacy of FTCPNPs as PTAs for tumor ablation. We explored the heating properties of various components based on the photothermal properties of FTCPNPs, including FTCPNPs, Fe^III^-TA, TA, FeCl_3_ aqueous solution and free Ce6 solution at the same concentration. In contrast to the negligible variation observed for the other 3 compositions, only FTCPNPs and Fe^III^-TA complex showed a significant temperature rise (Fig. 2C). The results above demonstrated that the photothermal effects of FTCPNPs were highly correlated with Fe^III^-TA coordination. Moreover, the photothermal conversion efficiency (*η*) and photostability of NPs are essential factors in tumor photothermal treatment. FTCPNPs produced no discernible attenuation of their photothermal effects during 5 laser on-off irradiation cycles in this study, indicating their high photothermal stability for use as PTAs in the treatment of tumors (Fig. 2E). According to the maximum temperature change (∆T_Max_) and the time constant for heating transfer (*τ*_*s*_), the *η* of the FTCPNPs was calculated to be 35.65% under irradiation by 808 nm laser (Fig. 2F, G). According to previous reports, FTCPNPs had a higher photothermal conversion efficacy than Au (21%), Cu_2 − x_Se (22%), and Cu_9_S_5_ (26%), indicating a high photothermal conversion efficiency [34].

### Intracellular uptake of FTCPNPs and ROS generation by PDT

To enhance the phototherapeutic efficacy of FTCPNPs, their intracellular uptake was essential. Therefore, the intracellular uptake behavior of the FTCPNPs was investigated by CLSM. As expected, the fluorescence intensity in C918 cells increased with the extension of co-incubated time. After 6 h co-incubation, noticeable red fluorescence of FTCPNPs (from Ce6) was observed in C918 cells (Fig. 2H). The intracellular uptake of FTCPNPs was also analyzed quantitatively using flow cytometry. The results were consistent with CLSM observations, indicating that FTCPNPs have an affinity for tumor cells (Fig. 2I).

**Fig. 3:**
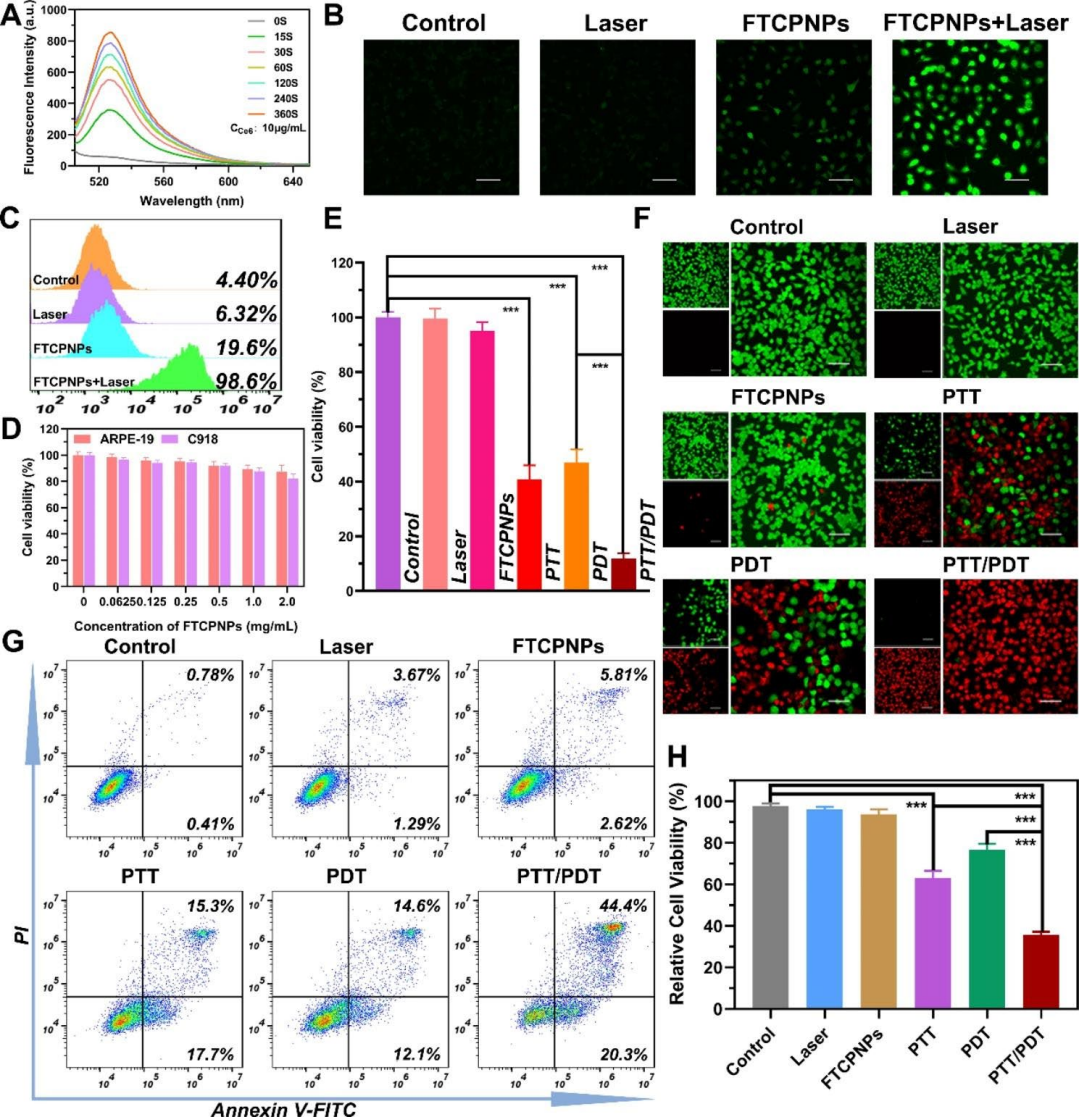
**A** Time-dependent ^1^O_2_ generation of FTCPNPs was irradiated by 660 nm laser (5 mW/cm^2^). The concentration of Ce6 was 10 µg/mL. **B, C** Following different treatments (660 nm laser dose: 5 mW/cm^2^, 3 min), DCFH-DA staining in C918 cells was observed by CLSM, and flow cytometry analysis was used to quantify ROS production. Scale bar: 100 μm. **D** ARPE-19 and C918 cell viability after co-incubated with FTCPNPs (n = 5). **E** C918 cell viability after various treatments (808 nm, 2.0 W/cm^2^, 5 min for PTT and 660 nm, 5 mW/cm^2^, 3 min for PDT). **F** After various treatments, CLSM images of C918 cells were stained with CAM and PI. Scale bar: 100 μm. **G, H** C918 cell apoptosis was analyzed by flow cytometry and quantitative analysis of each group

The premise of PDT is to effectively produce ROS under NIR excitation [35]. Therefore, a high level of ROS production is necessary for PDT to kill tumor cells effectively. SOSG is an indicator of the ^1^O_2_ level in vitro. After obtaining FTCPNPs (Ce6: 10 µg/mL) and exposure to 660 nm laser irradiation (5 mW/cm^2^), the fluorescence intensity of SOSG increased in a time-dependent manner (Fig. 3A). Moreover, under different irradiation durations, various concentrations of Ce6 (2, 4 and 8 µg/mL) showed a time-dependent increase in fluorescence (Fig. S3A, B, C).

The Ce6 in the FTCPNPs could be utilized as a photosensitizer for ROS production induced by PDT. DCFH-DA was used to determine the ROS levels after incubation with FTCPNPs. This ROS indicator emitted green fluorescence under CLSM observation when converted to 2′,-7′-dichlorofluorescein (DCF). The FTCPNPs showed apparent green fluorescence after 660 nm laser irradiation (Fig. 3B). The flow cytometry quantitative results agreed with the CLSM findings mentioned above (Fig. 3C). The control and laser groups exhibited negligible fluorescence (4.40% and 6.32%). The fluorescence intensity of the FTCPNPs group was 19.6%. As shown in Fig. 3C, upon 660 nm laser irradiation, C918 cells treated with FTCPNPs displayed evident green fluorescence (98.6%). The results demonstrated that FTCPNPs can generate considerable ROS in C918 cells under 660 nm laser irradiation and provide a theoretical foundation for PDT.

### In vitro synergistic therapeutic efficacy

The cytotoxicity of FTCPNPs against C918 cells was assessed using the traditional CCK-8 protocol following confirmation of the photothermal/photodynamic effects of FTCPNPs. Before that, ARPE-19 cells were co-incubated with different concentrations of FTCPNPs (0.0625, 0.125, 0.25, 0.5, 1.0 and 2.0 mg/mL) for 48 h, as determined by CCK-8 assay. Even at 2.0 mg/mL, there was still an excellent level of ARPE-19 cell viability (Fig. 3D). This study indicates that FTCPNPs are biologically safe and have the potential to be used in the field of medicine in the future. Then, to verify the efficacy of synergistic therapies, after various treatments, the viability of the cells changed in a concentration-dependent manner (FTCPNPs concentration: 0.5 and 1.0 mg/mL), suggesting that FTCPNPs at high concentrations exhibited more significant toxicity (Fig. 3E and Fig. S4). At the identical concentration of FTCPNPs (0.5 mg/mL), 40.86 ± 5.14 and 46.99 ± 4.84% of cells in the PTT group and PDT group survived, respectively, and only 11.91 ± 2.02% of the C918 cells survived in the PTT/PDT group which showed the most significant killing effect. Compared to the control group, PTT group and PDT group, significantly lower cell viability was observed in the PTT/PDT group. As a result, synergistic therapy was more effective than monotherapy (PTT or PDT-only) in achieving lethality. Subsequently, to more intuitively display the synergistic phototherapy performance of FTCPNPs, live/dead cell staining with fluorescence showed comparable cytotoxicity to C918 cells. As shown in Fig. 3F in the PTT/PDT group, almost all C918 cells died and fluoresced bright red, which further proved the excellent synergistic therapeutic efficacy of FTCPNPs. Additionally, flow cytometry was used to quantify apoptosis after various treatments (Fig. 3G, H), and the results agreed with the CCK-8 assay results.

**Fig. 4:**
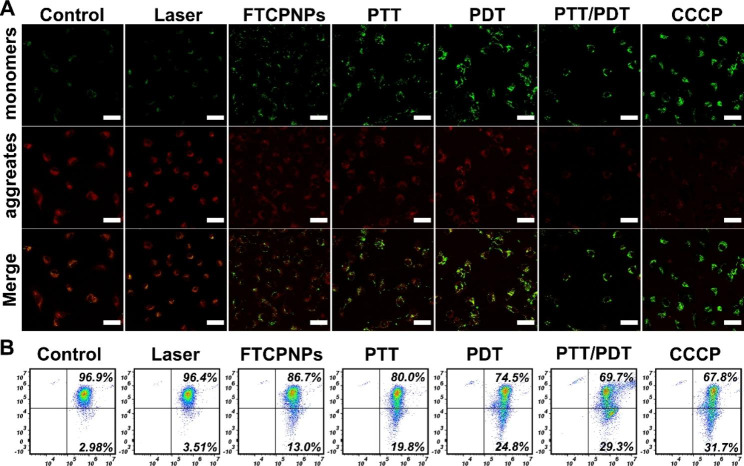
** A** Changes in MMP were observed with CLSM after different treatments. C918 cells treated with CCCP were used as the positive control group. Scale bar: 50 μm. **B** Measurement of mitochondrial depolarization using flow cytometry based on JC-1 assay

Damage to mitochondria can activate the intrinsic apoptosis pathway directly in cancer cells, which regulates life and death. In fact, various endogenous and exogenous stimuli can cause mitochondrial-dependent damage, including oxidative stress, ischemia, and DNA damage [36]. The role of ROS in cell metabolism is well-known, but high levels of intracellular ROS may cause mitochondrial damage, leading to mitochondrial-damage-dependent apoptosis [37]. Numerous studies have demonstrated that NIR-mediated PTT/PDT induces ROS, which activates oxidative stress and mitochondrial damage in cancer cells [38]. Therefore, to more intuitively observe the deterioration of C918 cell MMP induced by FTCPNPs under different treatments, we used CLSM to observe the change in membrane potential. As MMP decreases, JC-1 probe fluorescence converts from red to green. The group treated with CCCP exhibited strong green fluorescence due to JC-1 monomers. In Fig. 4A, it was found that synergistic therapy led to the most evident contrast of green-red fluorescence despite a single therapy method that induced apoptosis and lowered the MMP, thus emitting green fluorescence. Flow cytometry was used to quantify JC-1 aggregates and monomers. There was no significant change in the FTCPNPs group due to the weak chemodynamic therapy (CDT) effect, and the value of MMP in the PTT/PDT group decreased to 29.3% compared with control group (Fig. 4B). These results confirm that FTCPNPs damaged the integrity and function of mitochondria which showed that the C918 cells treated with the FTCPNPs were apoptosis by the mitochondrial apoptosis pathway under NIR laser irradiation(880 nm and 660 nm).

**Fig. 5:**
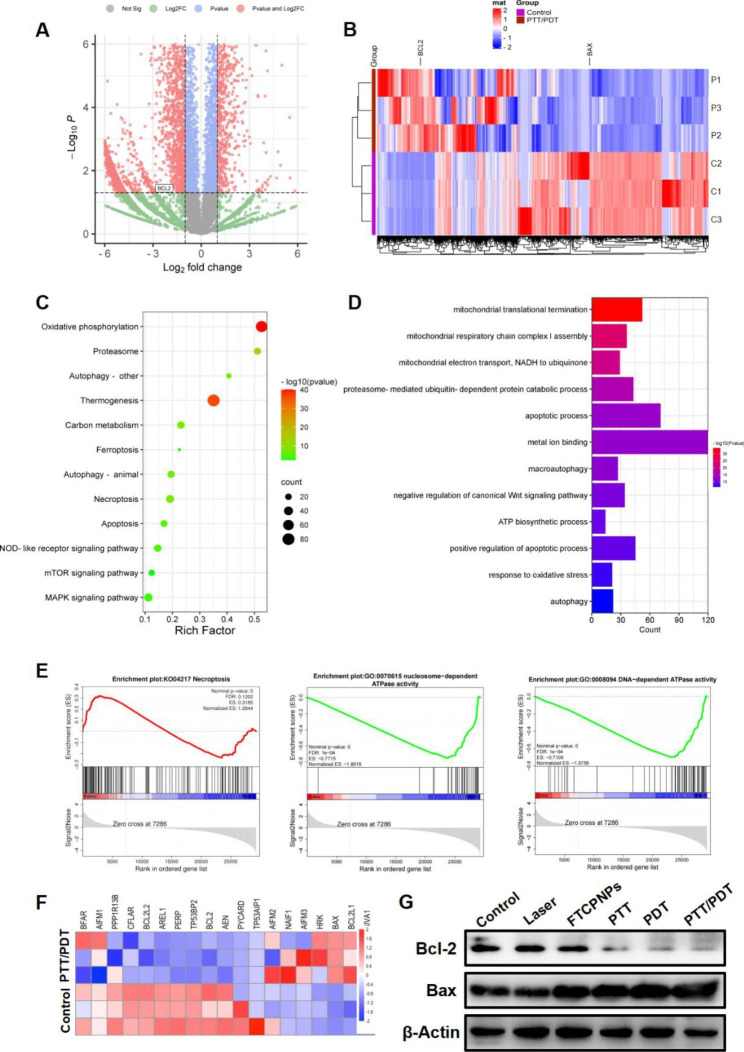
**A** The volcano map showed gene expression changes in C918 cells after treatment. **B** Heat map showed the differentially expressed genes after clustering between the control group and the PTT/PDT group. **C** KEGG analysis showed significantly enriched pathway changes after co-treatment, such as autophagy, apoptosis, and thermogenesis, etc. **D** GO showed changes in biological function, such as apoptosis and mitochondrial function, after synergistic therapy. **E** GSEA showed enriched Necroptosis and ATPase activity pathways. **F** Heat map showed the differential changes in apoptosis-related genes after synergistic treatment. **G** Western-blot showed the changes of apoptosis-related genes Bcl-2 and Bax

Then, we examined the mRNA profiling in C918 cells treated with PBS (Control) and FTCPNPs + 808 nm and 660 nm laser (PTT/PDT) by RNA-sequencing to explore the potential therapeutic mechanism. Both the control and PTT/PDT groups contained thousands of mRNA transcripts, including 2399 up-regulated genes and 4924 down-regulated genes (Fig. 5A). Cluster heat map of differential gene expression upon treatment were shown in Fig. 5B. Additionally, KEGG pathway enrichment analysis revealed that DEGs were significantly enriched in 12 pathways, including apoptosis, autophagy and thermogenesis (Fig. 5C). Similarly, GO analysis also showed changes in biological functions such as apoptosis and mitochondrial dysfunction (Fig. 5D). GSEA analysis further enriched the pathways related to apoptosis and mitochondrial function (Fig. 5E), suggesting that PTT/PDT synergistic treatment caused mitochondrial dysfunction and apoptosis in C918 cells. To further analyze the relationship between PTT/PDT treatment and apoptosis, we analyzed the apoptosis-related genes after PTT/PDT treatment. We found that most apoptosis-related genes were significantly up-regulated after PTT/PDT treatment (Fig. 5F). Then we used Western-blot to verify the changes in the expression of the apoptosis-related core molecules Bcl-2 and Bax. As expected, Bcl-2 was significantly down-regulated and Bax was significantly up-regulated after treatment (Fig. 5G). Thus, these results suggested that FTCPNPs treatment induces mitochondrial damage through PTT/PDT and promoted C918 cell apoptosis.

### Dual-modal imaging and bio-distribution of FTCPNPs

**Fig. 6:**
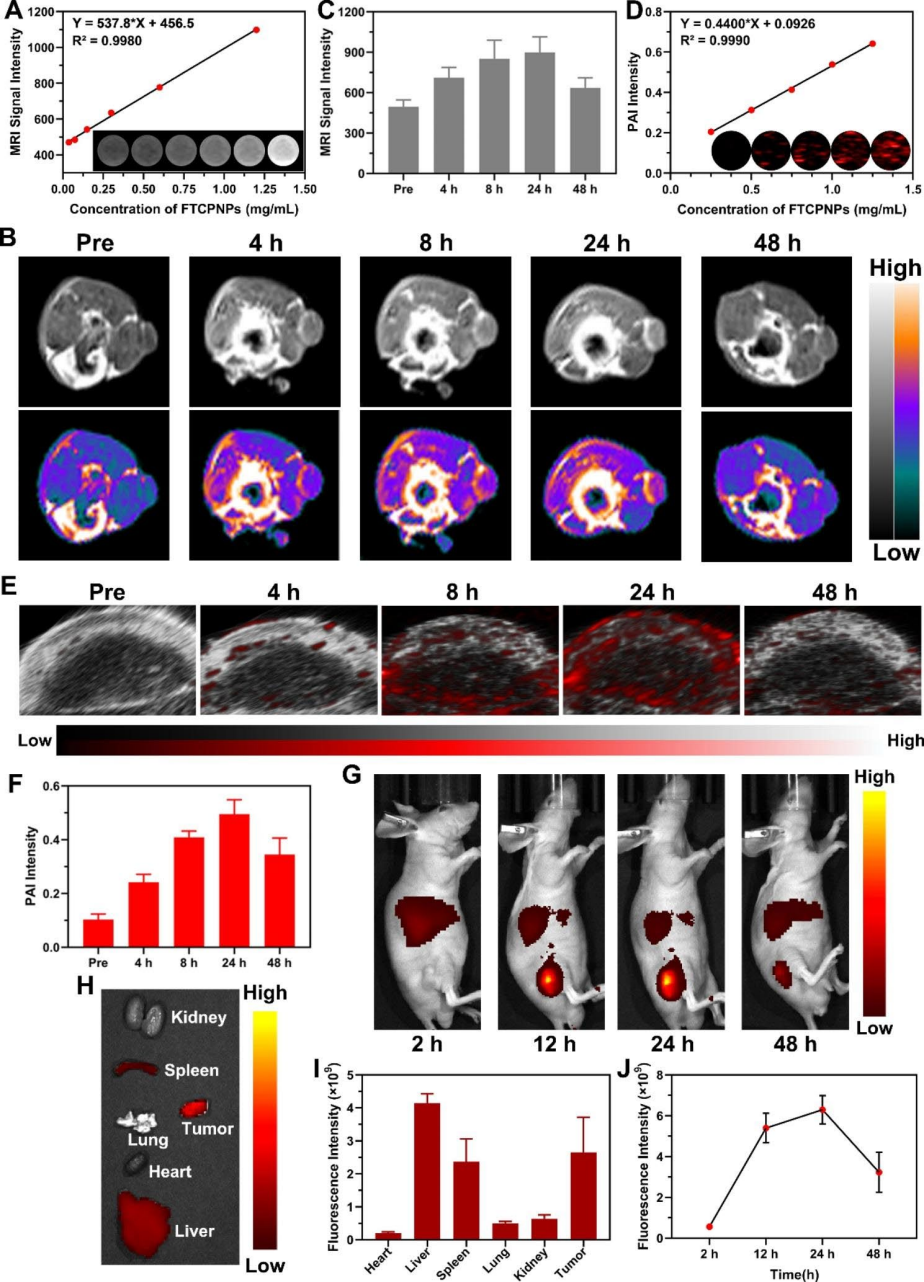
**A** The T1 signal intensity and FTCPNPs concentration (0.0375, 0.075, 0.15, 0.3, 0.6 and 1.2 mg/mL) were linearly related. T1-weighted MRI images of FTCPNPs aqueous solution at various concentrations were shown in the inset. **B** After intravenous injection of FTCPNPs, time-dependent T1-weighted MRI images of C918-tumor-bearing mice were obtained and **C** the intensity of the corresponding MR signal (n = 3). **D** The linear correlation of PA signal intensity and the concentration of FTCPNPs (0.25, 0.5, 0.75, 1.0 and 1.25 mg/mL) and the PAI images of FTCPNPs aqueous solution at various concentrations were shown in the inset (λex = 690 nm). **E** Tumor-region PAI images in vivo and **F** the corresponding signal intensity values (n = 3). **G** After intravenous injection of FTCPNPs, FL images of C918-tumor-bearing mice were obtained. **H** Ex vivo FL images of major organs and tumor dissected from mice 48 h after FTCPNPs injection. **I** Analysis of the quantitative bio-distribution of FTCPNPs as measured by the average FL signal intensity of organs and tumors in mice (n = 3). **J** Quantitative analysis of FL signal intensity with time in the tumor region (n = 3)

In the clinic, MRI is one of the most prevalently used imaging modalities for diagnostic purposes due to its high spatial resolution and deep tissue penetration [39]. Due to the excellent T1-MRI capability of Fe^III^ [40], FTCPNPs are expected to be contrast-enhanced agents in clinical applications. Therefore, in vitro and in vivo imaging performances of FTCPNPs were systematically evaluated. In this study, FTCPNPs were shown to be effective for T1-MRI, with an excellent linear relationship between MR signal intensity and the concentration of FTCPNPs (Fig. 6A). Then, FTCPNPs were intravenously injected into C918-tumor-bearing mice to evaluate the capability of MRI in vivo. The T1 signal of the tumor region increased with time, peaked at 24 h and then decreased (Fig. 6B). At 24 h post-injection, the quantitative analysis of T1 signal intensity peaked (Fig. 6C).

PAI, as a new tumor imaging method with high sensitivity and spatial resolution, can show the accumulation of nanoagents in the tumor region, and adjust the treatment time window and therapeutic response [41]. The high NIR photothermal conversion efficiency of FTCPNPs also motivated us further to investigate the potential of FTCPNPs as PAI contrast agents. Thus, the dispersion of the FTCPNPs was subjected to full wavelength (680 − 970 nm) scanning under the PAI system. The highest PA intensity of the FTCPNPs was located at 690 nm, which was used as the excitation wavelength (Fig. S5). As expected, the PA signal of FTCPNPs was evidently concentration-dependent and linearly related to concentration (Fig. 6D). The FTCPNPs were then evaluated in vivo for PAI. After approximately 24 h, the tumor regions showed the highest PA signal intensity (Fig. 6E), corresponding to the time of maximum signal intensity in MRI. According to quantitative analysis of PA signal intensity measurements, a change in PA signal intensity was detected during the 48 h (Fig. 6F). Accordingly, FTCPNPs hold great promise for biomedical applications as an efficient MRI/PAI contrast agent.

FTCPNPs can be further evaluated by fluorescent (FL) imaging to determine tumor accumulation and bio-distribution. DiR fluorescence-labeled FTCPNPs were injected into C918-tumor-bearing nude mice and FL imaging was performed at different time points. In Fig. 6G, time-dependent FL signals were observed within the tumor region. FL signals at the tumor region peaked 24 h after injection and significantly decreased at 48 h. These results were consistent with MRI and PAI. Subsequently, the distribution of FTCPNPs in mice’s main organs and the tumorswere detected (Fig. 6H). As a result of phagocytosis of the RES, the liver and spleen accumulated FTCPNPs, which were metabolized in the body according to the normal physiological pathway. Both in vitro and in vivo FL imaging trends were consistent. There was evidence that FTCPNPs could accumulate effectively in tumor tissue for an extended period without causing damage to major organs (Fig. 6I). A fluorescence analysis system was then used to quantify the time corresponding to the fluorescence intensity (Fig. 6J).

### In vivo anticancer efficacy under PTI guidance and biosafety of FTCPNPs

**Fig. 7:**
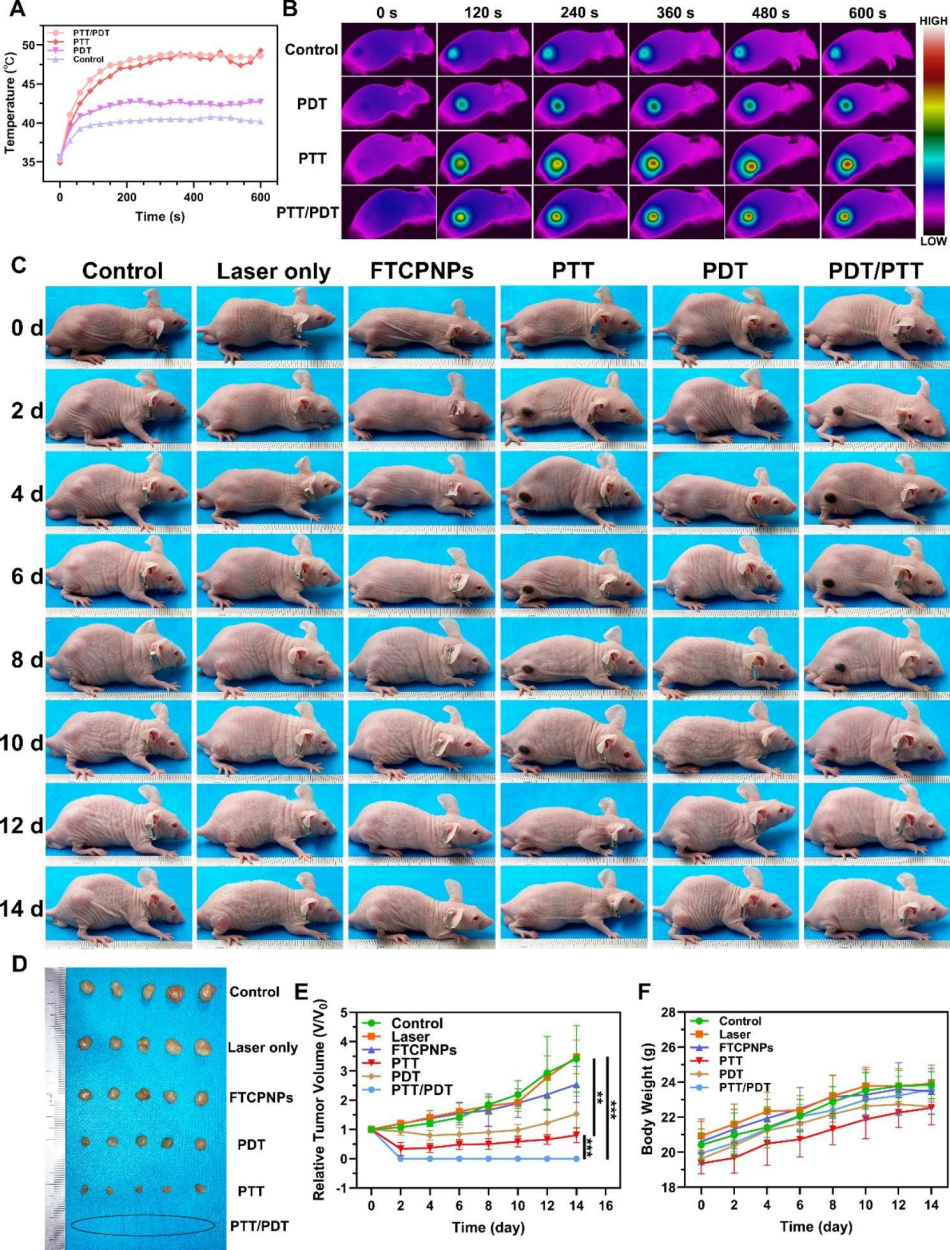
** A** Temperature change curve of the tumor site in C918-tumor-bearing mice and **B** corresponding infrared thermal images (808 nm laser doses: 2.0 W/cm^2^, 10 min; 660 nm laser dose: 95 mW/cm^2^). **C** Digital pictures of C918-tumor-bearing mice of each group during 14 d observation after different treatments. **D** Tumor dissection photographs from 6 groups of mice. **E** Time-dependent curves of the relative growth of tumor volume among each group. **F** Time-dependent curves of mice body weight among each group

According to the PAI, MRI, and FL imaging results, the optimal time for tumor treatment is 24 h after injection. Consequently, PTI was performed to monitor the therapeutic process in vivo 24 h after intravenous injection of FTCPNPs. After 808 nm (2.0 W/cm^2^, 10 min) and 660 nm (95 mW/cm^2^, 10 min) laser irradiation, the temperature in the tumor region rose rapidly to 48.6 °C, exceeding the threshold for tumor ablation. The temperature was controlled under 43 ℃ in the PDT group and the thermal camera monitored the treatment process in real-time to prevent PTT effects (Fig. 7A). Representative PTI images of each group of mice were presented (Fig. 7B), and the results provided sufficient information for monitoring the process of PTT. Compared with group (a), group (b) showed a minimal inhibitory effect, demonstrating that simple laser irradiation had negligible antitumor effects (Fig. 7C). A slight tumor inhibitory effect was observed in group (c), which may be due to the inhibitory effect of CDT. Furthermore, at 14 d post-injection, PTT in group (d) and PDT in group (e) resulted in a moderate reduction in tumor volume compared to the control group. Interestingly, group (f) experienced a thorough eradication of tumors compared to the initial conditions (Fig. 7E). The enhanced therapeutic efficacy of FTCPNPs may be attributed to their combination of PDT and PTT effects. Consistently, visualization of tumors in vitro followed a corresponding trend (Fig. 7D). Compared to the PTT or PDT-only group, FTCPNPs had a more significant synergistic therapeutic effect on tumor inhibition rate in the PTT/PDT group (Fig. S6). Similarly, tumor weights excised from mice 14 d after treatment followed a similar pattern as tumor volume (Fig. S7). Additionally, the mice showed negligible changes in body weight in each group, demonstrating the excellent biocompatibility of the FTCPNPs and the tolerability of the dose used in the research (Fig. 7F).

**Fig. 8:**
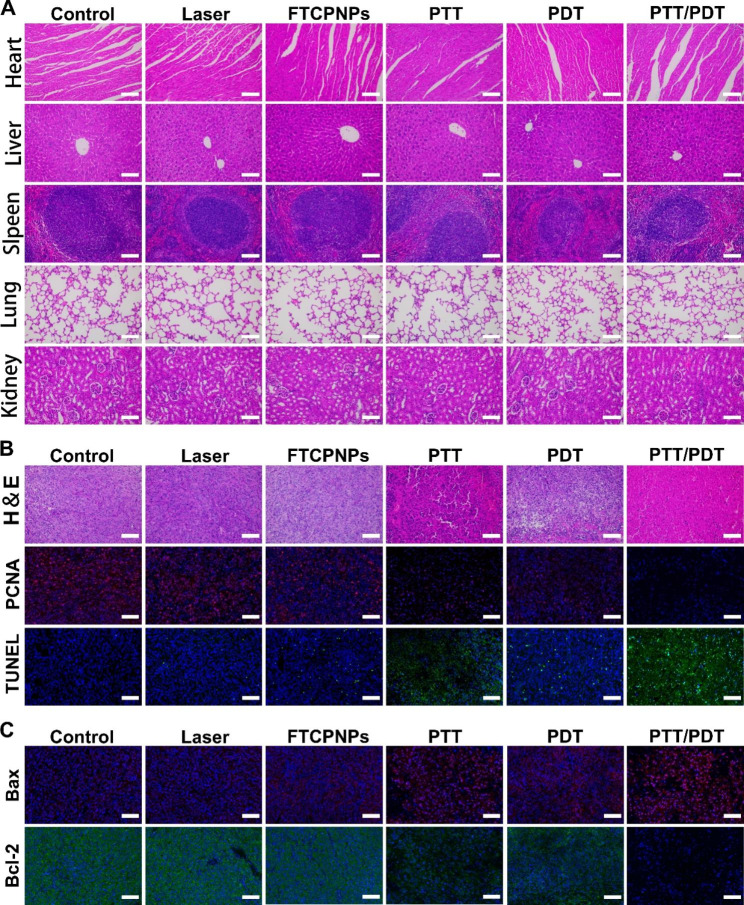
** A** Major organs (heart, liver, spleen, lung, kidney) were stained with H&E in each group. Scale bar: 100 μm. **B** Staining of tumors with H&E, PCNA and TUNEL in each group. Scale bar: 100 μm. **C** Tumors were collected from each group on the first post-treatment day with Bax and Bcl-2 immunofluorescence staining. Scale bar: 100 μm

In this study, H&E was used to stain the major organs (heart, liver, spleen, lung, kidney) at 24 h post-treatment. They demonstrated negligible toxicity in each group in vivo (Fig. 8A). H&E, PCNA, and TUNEL staining of the tumor region also verified that the killing effect of the co-treatment group was more evident than that of the other treatment groups (Fig. 8B). Moreover, immunofluorescence staining images showed that FTCPNPs under excitation with 808 nm and 660 nm laser could increase the expression of Bax and inhibit Bcl-2 effectively. These studies demonstrated that the FTCPNPs could effectively induce apoptosis of C918 cells through the mitochondrial pathway in vivo (Fig. 8C). This was consistent with the previous Western-blot results.

To assess the biological safety of FTCPNPs, routine blood test and blood biochemical analyses were conducted on healthy Kunming mice. At 28 d after treatment, all FTCPNP-treated groups showed negligible differences from the control group (Fig. S8A). H&E staining of major organs from healthy Kunming mice showed no detectable damage at any concentration in vivo (Fig. S8B). After PTT/PDT combination therapy, the FTCPNPs showed significant antitumor effects and negligible systemic toxicity.

## Conclusions

Overall, an unlimited potential multifunctional nanoplatform (FTCPNPs) was successfully constructed for MR/PA dual imaging-guided PTT/PDT for the treatment of UM. Notably, under the excitation of 808 nm laser, FTCPNPs can generate local heat to ablate tumors, which has excellent photothermal effects and photothermal conversion efficiency. The *η* of FTCPNPs was calculated to be 35.65%, and the FTCPNPs could perform in vivo treatment under the guidance of PTI. Moreover, the Ce6 component initiated the PDT process, produced cytotoxic ^1^O_2_, and then induced tumor cell apoptosis by damaging mitochondria, which enhanced the therapeutic effect of PTT. At the same time, the NPs can realize MR/PA bimodal imaging of C918 subcutaneously transplanted tumors in nude mice. At last, through systematic evaluation and determination of its therapeutic effect, they preliminarily proved that the NPs had good biological safety in vivo and in vitro. As a result, this study presented a synergistic PTT/PDT nanoprobe for tumor detection and phototherapy treatment, which offered a potential approach toward developing an ideal method for treating UM.

## Electronic supplementary material

Below is the link to the electronic supplementary material.

Supplementary Material 1

## Data Availability

All data analyzed during this study are included in this published article and its supplementary information files.

## Abbreviations

UM: uveal melanoma
MRI: magnetic resonance imaging
MR: magnetic resonance
PAI: photoacoustic imaging
PA: photoacoustic
PTAs: photothermal agents
NIR: near-infrared
ROS: reactive oxygen species
PTT: photothermal therapy
PDT: photodynamic therapy
FTCPNPs: Fe^III^-TA/PLGA/Ce6 nanoparticles
CPNPs: PLGA/Ce6 nanoparticles
Ce6: chlorin e6
CH_2_Cl_2_: dichloromethane
PLGA: poly-lactic-co-glycolic acid
FDA: Food and Drug Administration
PVA: polyvinyl alcohol
Fe^III^-TA: Fe^III^-tannic acid
^1^O_2_: singlet oxygen
TEM: transmission electron microscope
SEM: scanning electron microscopy
CLSM: confocal laser scanning microscope
ICP-OES: inductively coupled plasma optical emission spectrometer
VSM: vibrating sample magnetometer
C918: the human choroid melanoma cells
ARPE-19: adult retinal pigment epithelial cell line-19
DCFH-DA: 2′,-7′-dichlorodi-hydrofluorescein diacetate
SOSG: singlet oxygen sensor green
MMP: mitochondrial membrane potential
PMSF: phenyl methane sulfonyl fluoride
PVDF: polyvinylidene fluoride
CAM: Calcein-AM
PI: propidium iodide
DAPI: 4,6-diamidino-2-phenylindole
DiR: 1,1′-dioctadecyl-3,3,3′,3′-tetramethylindotricarbocyanine iodide
H&E: hematoxylin and eosin
TUNEL: transferase-mediated dUTP nick-end labeling
PCNA: proliferating cell nuclear antigen
HAADF: high-angle annular dark-field
PDI: polydispersity
RES: reticuloendothelial system
FL: fluorescent
