# Supplementary material for: Cooperative phototherapy based on bimodal imaging guidance for the treatment of uveal melanoma
Source: J Nanobiotechnology. 2023 May 4;21:146. doi: 10.1186/s12951-023-01891-6 (PMC10161622; doi:10.1186/s12951-023-01891-6)
Supplement: Supplementary file 1 — Supplementary Material 1 [file 12951_2023_1891_MOESM1_ESM.docx]

**Supporting Information**

**Cooperative phototherapy based on bimodal imaging guidance for the treatment of uveal melanoma**

Tong Huang^1^, Xinzhi Xu^2^, Chen Cheng^1^, Jianxin Wang^1,3*^ and Liping Yang^4*^

^1^Chongqing Key Laboratory of Ultrasound Molecular Imaging, The Second Affiliated Hospital of Chongqing Medical University, Chongqing, 400010, P. R. China.

^2^Department of Ultrasound, Chongqing University Cancer Hospital, Chongqing, 400030, P. R. China.

^3^Department of Ultrasound, The First Affiliated Hospital of Harbin Medical University, Harbin 150001, P. R. China.

^4^Department of Laboratory Medicine, The Second Affiliated Hospital of Chongqing Medical University, Chongqing, 400010, P. R. China.

Corresponding authors: Jianxin Wang and Liping Yang

E-mail addresses: wjxhmu@163.com (Jianxin Wang), 306843@hospital.cqmu.edu.cn (Liping Yang)

**Part A: Calculation formula**

**Encapsulation efficiency and loading capacity**

The encapsulation efficiency (EE) and loading capacity (LC) were calculated as follows:

$$\mathrm{EE}\left( \% \right)=\frac{Total amount of drug-amount of drug in supernatant}{Total amount of drug}\times100\%$$

$$\mathrm{LC}\left( \% \right)=\frac{Total amount of drug-amount of drug in supernatant}{\mathrm{Weight}\mathrm{of} dried nanoparticles}\times100\%$$

**Table S1** The concentration of FTCPNPs corresponds to the concentration of Fe and Ce6:

| FTCPNPs | Fe | Ce6 |
| --- | --- | --- |
| 0.0625 mg/mL | 0.4833 ± 0.02191 μg/mL | 2.850 ± 0.08099 μg/mL |
| 0.125 mg/mL | 0.9666 ± 0.04382 μg/mL | 5.701 ± 0.1620 μg/mL |
| 0.25 mg/mL | 1.933 ± 0.08763 μg/mL | 11.40 ± 0.3239 μg/mL |
| 0.5 mg/mL | 3.866 ± 0.1753 μg/mL | 22.80 ± 0.6479 μg/mL |
| 0.75 mg/mL | 5.799 ± 0.2629 μg/mL | 34.20 ± 0.9718 μg/mL |
| 1.0 mg/mL | 7.732 ± 0.3505 μg/mL | 45.61 ± 1.296 μg/mL |
| 1.25 mg/mL | 9.666± 0.4382 μg/mL | 57.01 ± 1.620 μg/mL |
| 2.0 mg/mL | 15.46 ± 0.7011 μg/mL | 91.21 ± 2.592 μg/mL |

**Photothermal conversion efficiency of FTCPNPs**

The photothermal conversion efficiency of FTCPNPs was measured according to the previous method [1]. Briefly, 200 μL FTCPNPs (1.0 mg/mL) solution in 96-well was irradiated under 808 nm laser at a power density of 2.0 W/cm^2^ for 10 min. Then the laser was turned off and the temperature change of the solutions was real-time recorded. The time constant (*τ_s_*) for heat transfer was determined by applying the linear time-dependent data collected during the cooling period. The photothermal conversion efficiency (*η*) was calculated by the following equations:

(1). *η*=$\frac{hS\Delta T_{\mathrm{Max}}-Q_{\mathrm{Dis}}}{I(1-{10}^{-A_{808}})}$

In this equation, *η* is the conversion efficiency value, ∆T_Max_ is the maximum temperature change when the temperature reaches a steady state. Q_Dis_ represents heat dissipated from the laser mediated by the solvent and container. I is the laser power density. Lastly, *A*_808_ is the absorbance of the solution at 808 nm in the UV-Vis spectrum. Only the value of *hS* was unknown and could be calculated from the following equation.

(2). *τ_s_*=$\frac{m\cdot C_{w}}{hS}$

m is the mass of the solution used to suspend the nanoparticles, C_w_ here is the specific heat capacity of the solution and *τ_s_* is the time constant for heat transfer. As a typical example, we calculated the *η* value of FTCPNPs in detail. Here, the mass of water (m) is 0.2 g, the heat capacity of water (C_w_) is 4.2 J/(g·℃), and *τ_s_* of FTCPNPs is determined to be 189.0 s from the linear time-dependent data collected during the cooling period as shown in Fig. 2F. According to equation (2), the *hS* is calculated to be 4.444444 × 10^-3^ W/℃.

For the sample of FTCPNPs, ∆T_Max_ value is 39.6 ℃. Q_Dis_ here is measured to be 8.888889 mW. I is the laser power density that is 2.0 W/cm^2^. *A*_808_ is the absorbance of FTCPNPs at 808 nm in the UV-Vis spectrum which is determined to be 0.116. According to equation (1), the photothermal conversion efficiency (*η*) of FTCPNPs was calculated to be 35.65%.

**References**

1. Xi D, Xiao M, Cao J, Zhao L, Xu N, Long S, Fan J, Shao K, Sun W, Yan X et al: NIR Light-Driving Barrier-Free Group Rotation in Nanoparticles with an 88.3% Photothermal Conversion Efficiency for Photothermal Therapy. Adv Mater 2020, 32(11):e1907855.

**Part B: Supplementary figure**

**
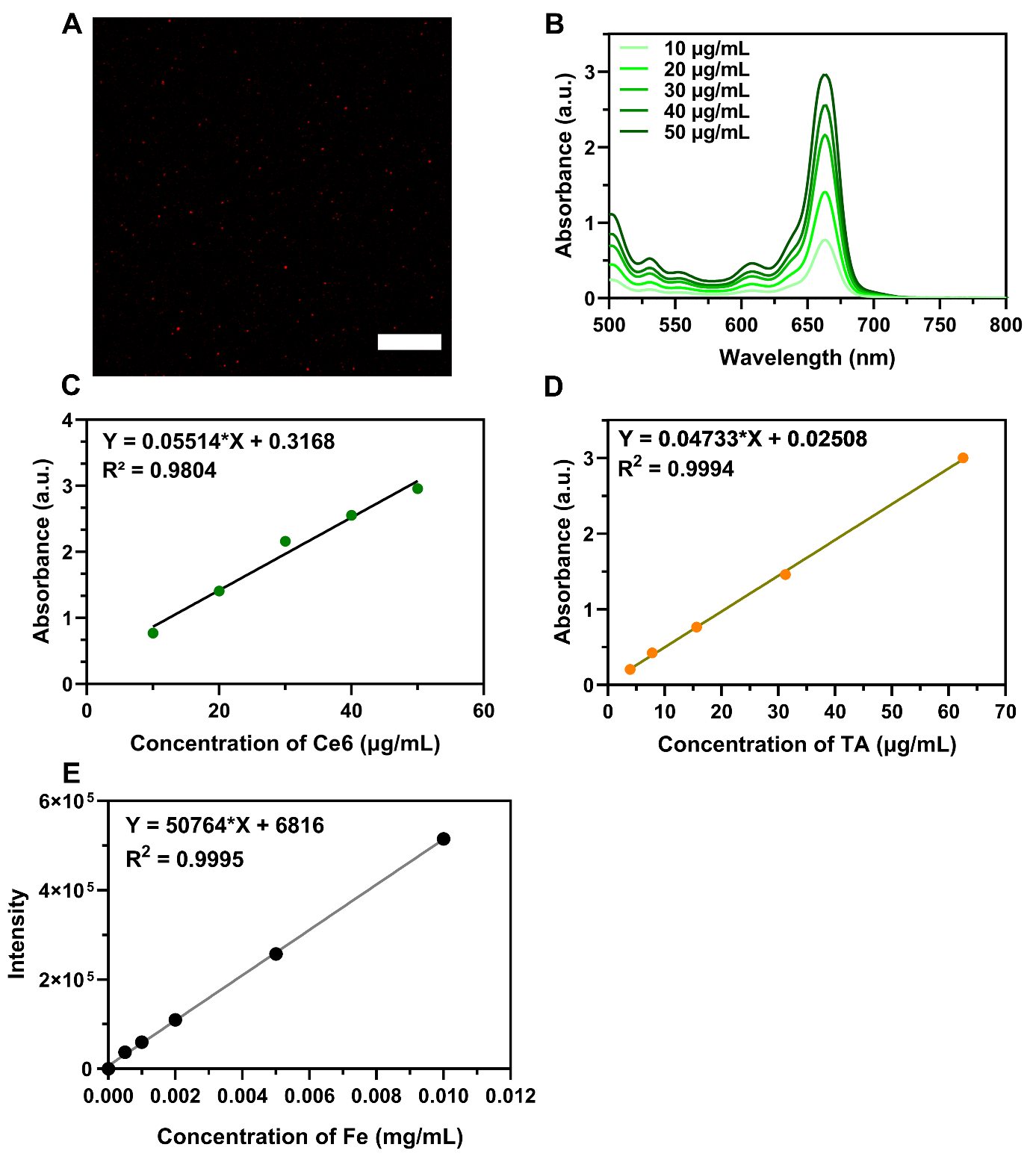
**

**Fig. S1 A** CLSM image of FTCPNPs (red fluorescence comes from Ce6). Scale bar: 50 μm. **B** UV-Vis absorption spectrum of free Ce6 with different concentrations and **C** the standard curve of free Ce6. **D** The standard curve of TA. **E** The standard curve of Fe

**Fig. S2** Infrared thermal images of FTCPNPs aqueous solution irradiated under increased power intensities (0.5, 1.0, 1.5, and 2.0 W/cm^2^)


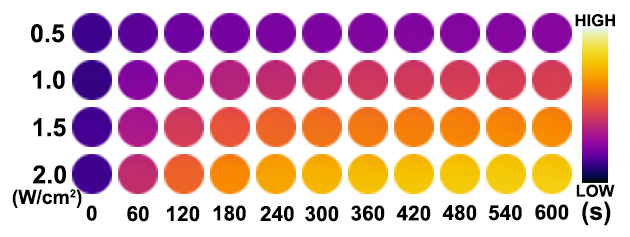


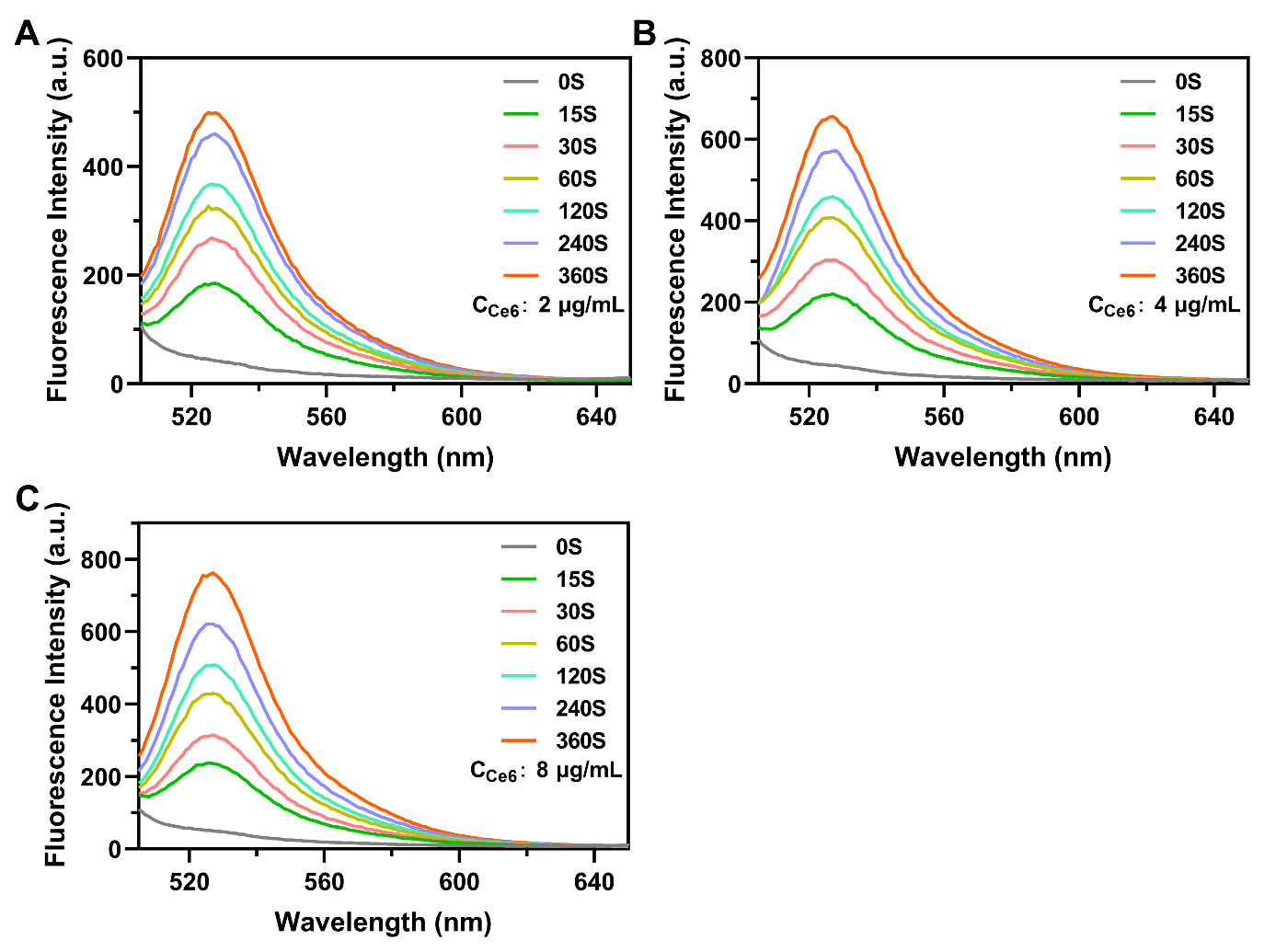


**Fig. S3 A, B, C** Time-dependent ^1^O_2_ generation of FTCPNPs at different concentrations irradiated by 660 nm laser (5 mW/cm^2^)


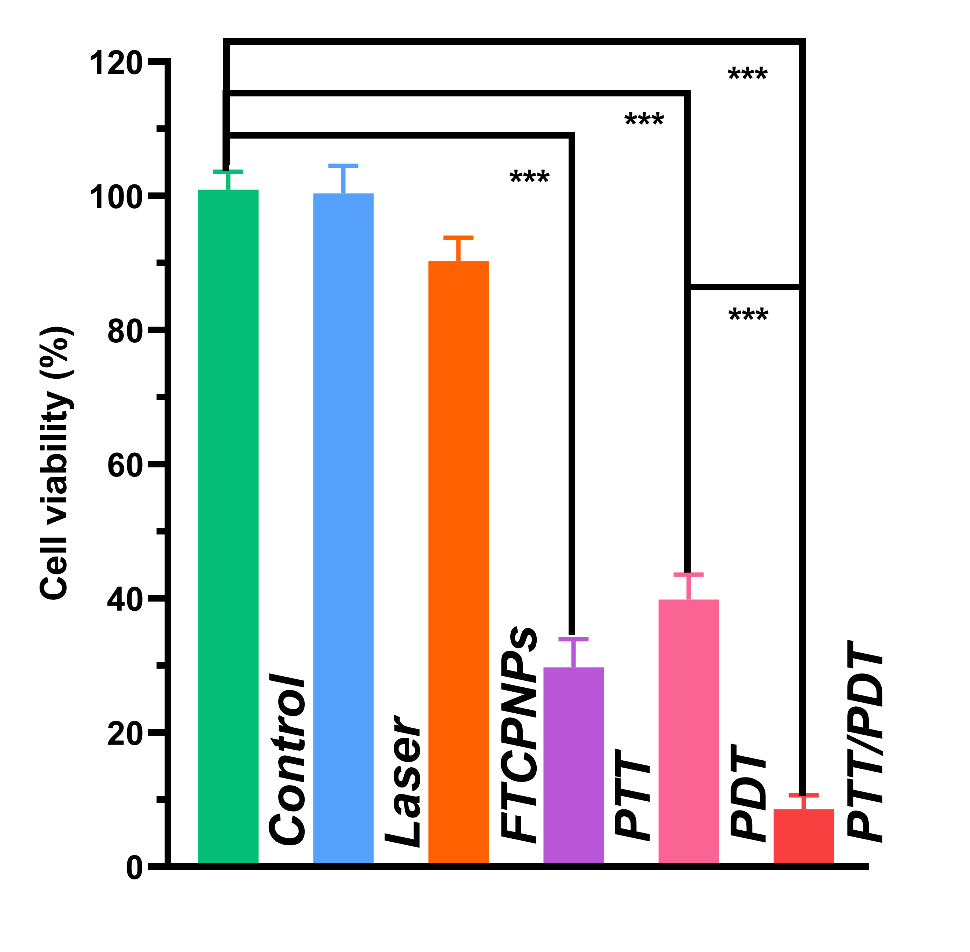


**Fig. S4** The cell viability after varied treatments (FTCPNPs concentration: 1.0 mg/mL) (n = 5)


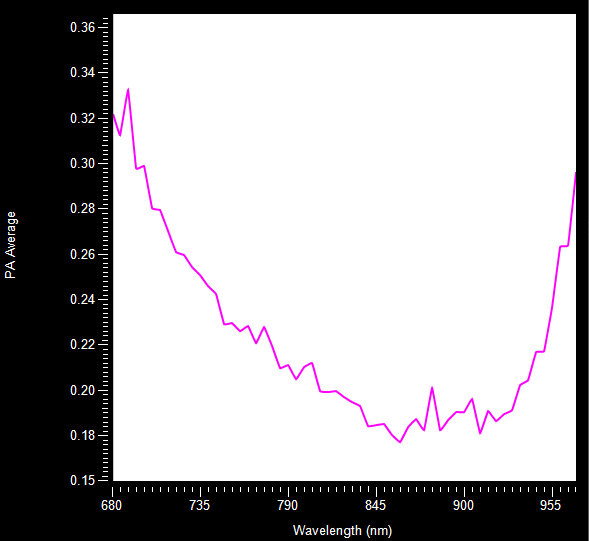


**Fig. S5** PA intensity under full-spectrum scanning of FTCPNPs


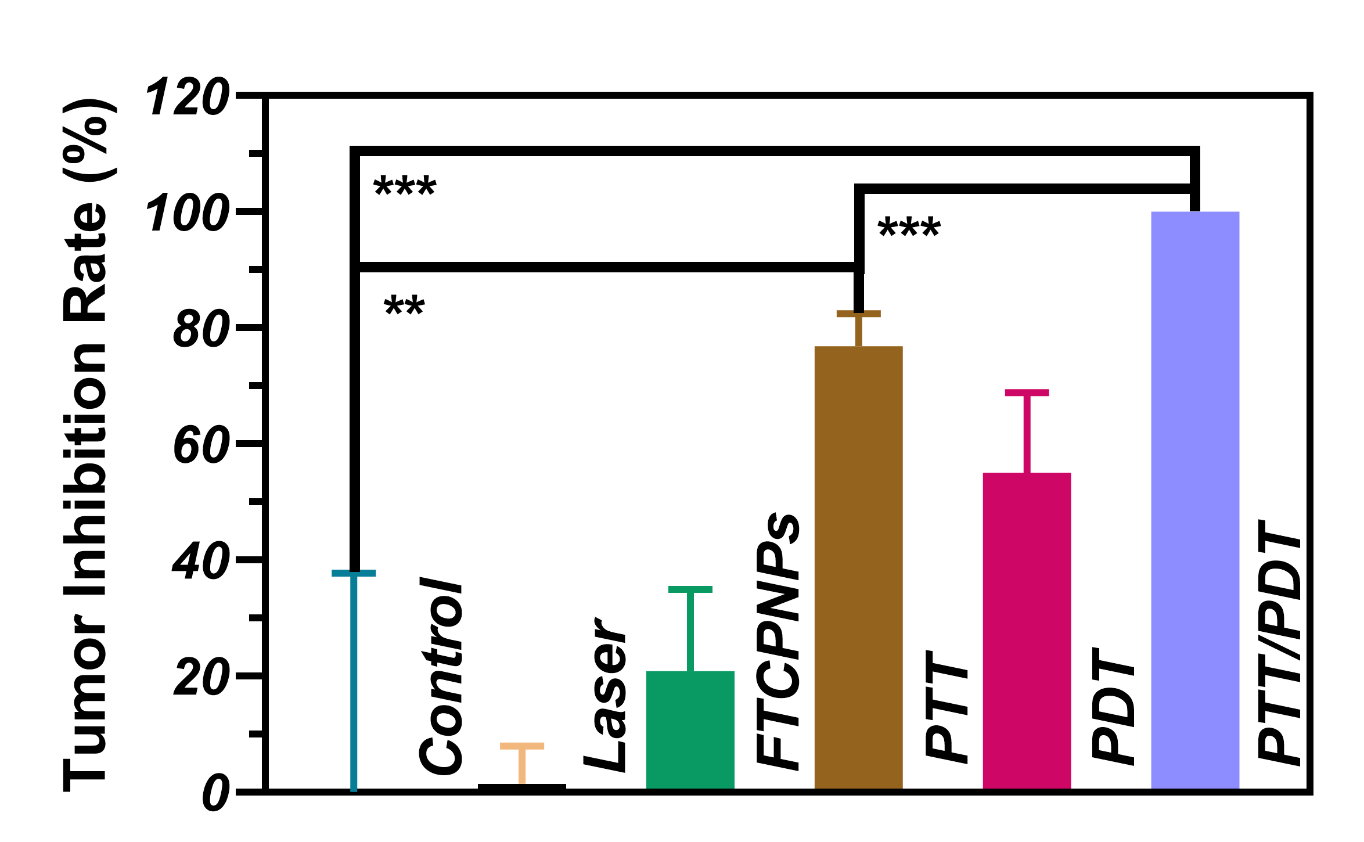


**Fig. S6** Tumor inhibition rate of C918-tumor-bearing nude mice after different treatments (n = 5, ***p < 0.001)


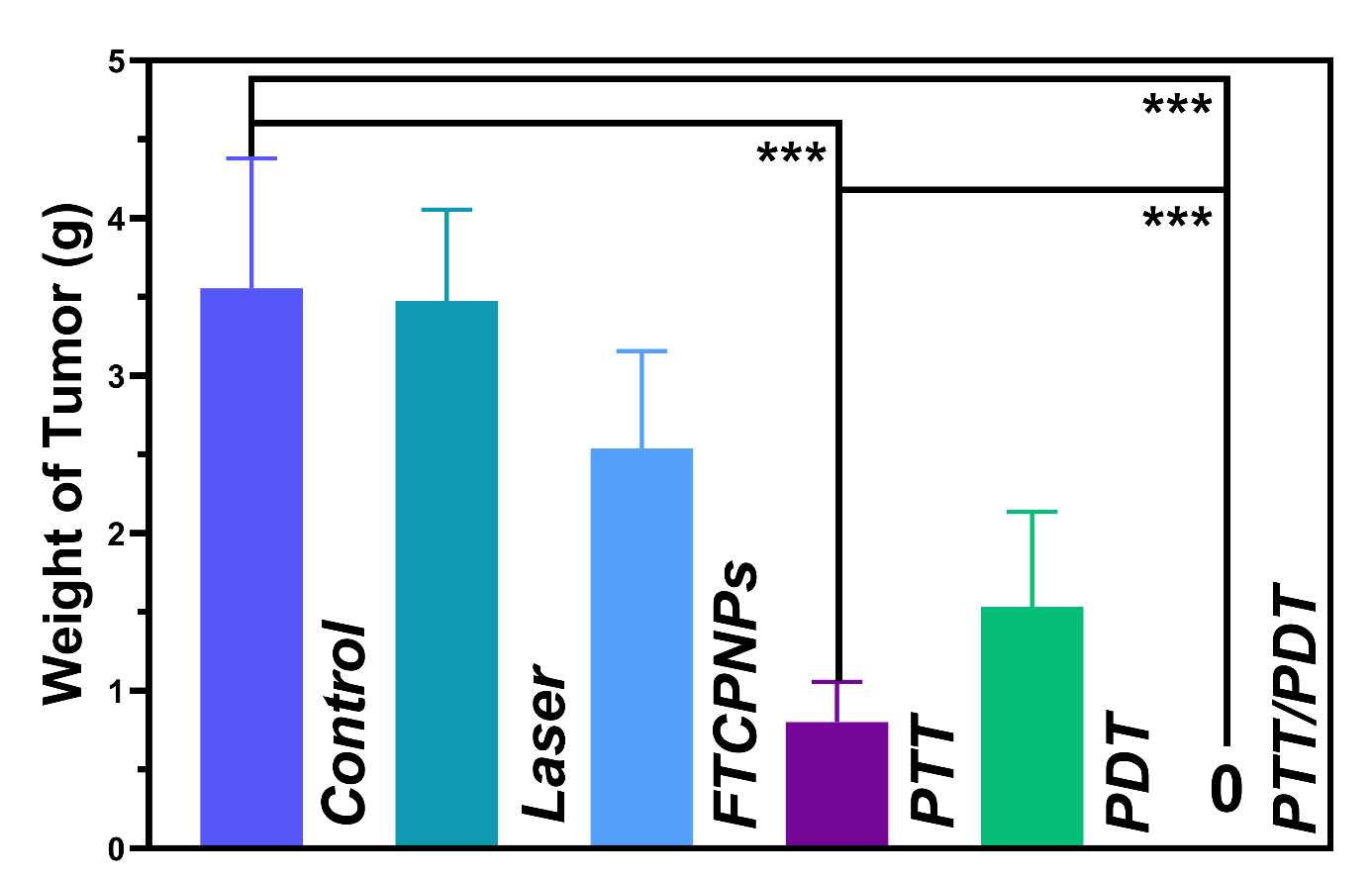


**Fig. S7** Tumor weight of different groups after 14 d of treatment (n = 5, ***p < 0.001)


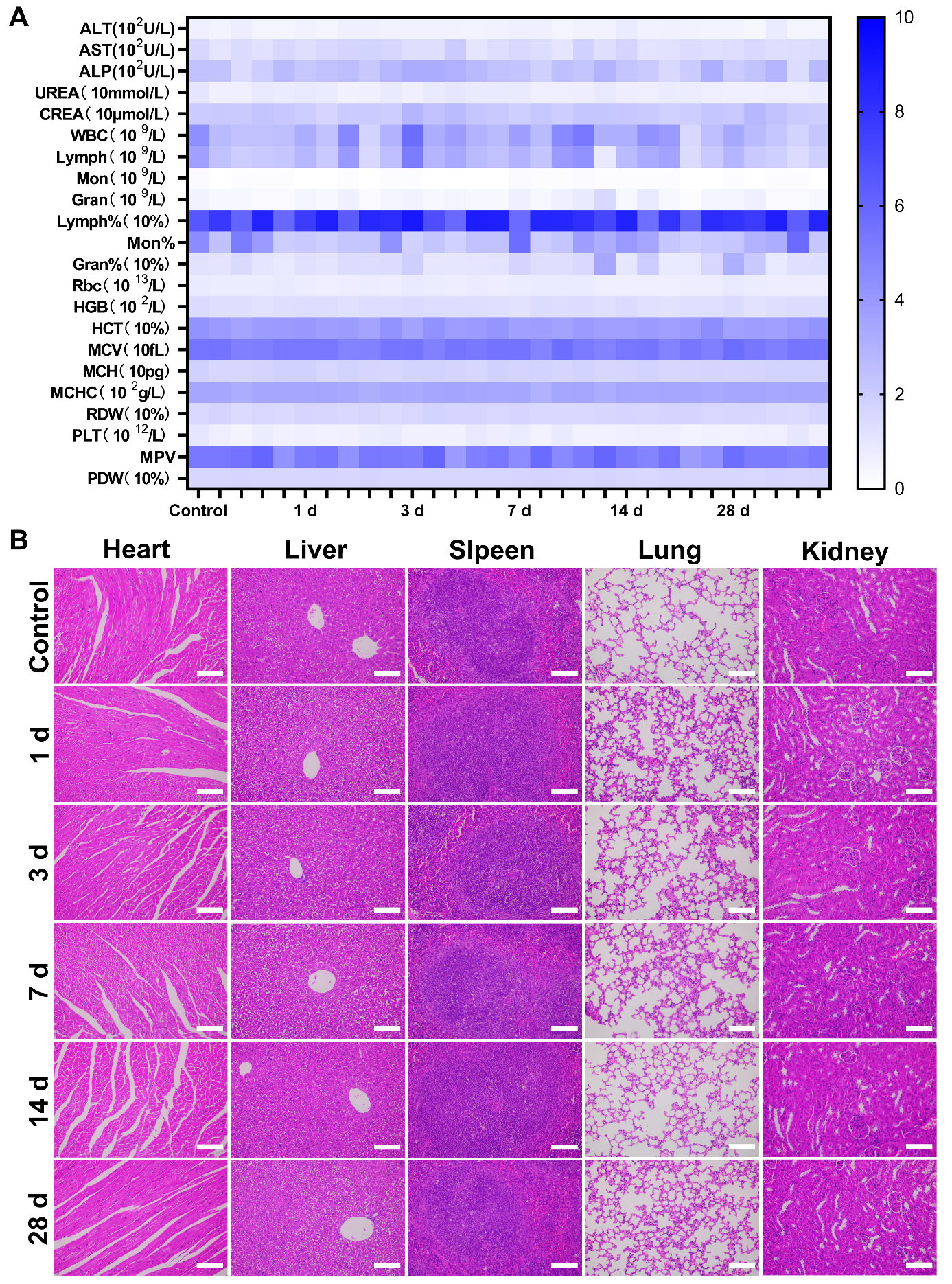


**Fig. S8 A** Routine blood and blood biochemical test of mice after intravenous injection of FTCPNPs (n = 5). **B** Images of H&E staining slices of major organs. Scale bar: 100 μm
